# Computational and *In silico* study of novel fungicides against combating root rot, gray mold, fusarium wilt, and cereal rust

**DOI:** 10.1371/journal.pone.0316606

**Published:** 2025-01-31

**Authors:** Mollah Naimuzzaman, Md Mahabub Hasan, Ajoy Kumer, Abu Yousuf Hossin, Mohammad Harun-Ur-Rashid, Swapan Kumar Roy, Abu Noman Faruq Ahmmed, Jamal Uddin

**Affiliations:** 1 College of Agricultural Sciences, IUBAT—International University of Business Agriculture and Technology, Dhaka, Bangladesh; 2 IUBAT Innovation and Entrepreneurship center, IUBAT—International University of Business Agriculture and Technology, Dhaka, Bangladesh; 3 Department of Chemistry, College of Arts and Sciences, IUBAT—International University of Business Agriculture and Technology, Dhaka, Bangladesh; 4 Department of Plant Pathology, Sher-e-Bangla Agricultural University, Dhaka, Bangladesh; 5 Department of Natural Sciences, Center for Nanotechnology, Coppin State University, Baltimore, Maryland, United States of America; University of Agriculture Faisalabad, PAKISTAN

## Abstract

The exploration of potential candidates for fungicides against four fungal proteins that cause some vital plant diseases, namely *Phytophthora capsici*, *Botrytis cinerea*, *Fusarium oxysporum* f. sp. *lycopersici*, and *Puccinia graminis* f. sp. *tritici*, was conducted using in silico, molecular docking simulations, and molecular dynamic (MD) simulation for selecting the nature of binding affinity with actives sites of proteins. First of all, the DFT was employed to optimize the molecular geometry, and get the prepared optimized ligand. From the DFT data, the chemical descriptors were calculated. Next, two docking tools, such as AutoDock by PyRx and Molecular Docking by Glide from the Schrödinger suite, were used to convey the docking score, and ligand protein interactions against four main proteases, for instance 7VEM, 8H6Q, 8EBB, and 7XDS having name of pathogens: *Phytophthora capsici*, *Botrytis cinerea*, *Fusarium oxysporum* f. sp. *lycopersici*, and *Puccinia graminis* f. sp. *tritici*, respectively. In case of auto dock from PyRx, the fungicides L01, L03, L04, L13, L14, L17, L18, and L19 demonstrated significantly higher affinities for binding to the four fungal pathogens. Surprisingly, it is conveyed that the L03 illustrated the highest binding score against three of 7VEM, 8EBB, and 7XDS proteins and L09 is highest for 8H6Q. However, MD was performed to check the validation and calculation the docking procedure and stability of the protein ligand docked complex accounting of RMSD, RMSF, SASA, Radius of gyration (Rg), Protein secondary structure elements (SSE), Ramachandran plot which confirm that the stability of docked complex is so high, and number of calculating the hydrogen bonds is more than good enough, as a result it is concluded the docking procedure is valid. Finally, Difenoconazole (L03) has been considered as the most promising antifungal drug evaluated from the studies.

## Introduction

The broad and negative impact of plant pathogenic fungi on agriculture, ecology, and food security around the world highlights the urgent need for the development of novel fungicides. These fungal diseases are responsible for considerable crop productivity and quality losses, endangering farmers’ livelihoods as well as the global food supply chain [1]. Furthermore, plant diseases induced by fungi contribute to economic instability in agricultural businesses [2], increase pesticide dependency [3], and harm the environment owing to excessive chemical use [4]. The emergence of plant pathogenic fungus exacerbates this problem. Many fungus species have evolved resistance to current fungicides, making conventional chemical treatments ineffective over time. This resistance not only reduces the efficacy of disease control measures, but also requires greater dosages or the use of several fungicides, raising the danger of environmental pollution and harm to non-target organisms [5].

Developing novel fungicides is therefore critical to addressing these difficulties. These innovative methods must be tailored to target fungal diseases with greater precision, reduce environmental impact, and overcome resistance mechanisms. Advanced technologies, including as nanotechnology, bio-based fungicides, and molecular biology techniques, are laying the groundwork for the next generation of fungicides [6]. These techniques allow researchers to design targeted treatments that disrupt fungal life cycles while causing minimum harm to beneficial microbes and the surrounding ecosystem. Furthermore, novel fungicides are critical to guaranteeing sustainable farming operations. By incorporating them into crop protection measures, farmers can minimize their reliance on standard chemical treatments and adopt more environmentally friendly practices. This transition is critical for preserving biodiversity, protecting soil health, and sustaining long-term productivity in agricultural systems [7]. However, the compelling necessity for innovative fungicides arises from the profound impact of plant pathogenic fungi [8], such as *Phytophthora capsici*(PDB ID: 7vem) [9], *Botrytis cinerea* (PDB ID: 8H6Q) [10], *Fusarium oxysporum* f. sp. *lycopersici* (PDB ID: 8EBB) [11], and *Puccinia graminis* f. sp. tritici (PDB ID: 7XDS) [12], on global agriculture. These fungal pathogens are responsible for notable crop damages and economic losses [13], with *Fusarium oxysporum* causing annual economic losses worldwide due to its detrimental effects on tomato production [14]. The escalating resistance to traditional fungicides and their environmental repercussions further emphasize the critical necessity for alternative approaches [15]. Recent progress in computational methodologies, such as molecular docking [16], Molecular Dynamics (MD) simulations [17], Density Functional Theory (DFT) [18], as well as in silico techniques, present promising avenues for the discovery and enhancement of novel fungicidal targets.

Fungal infections significantly impact food security and cause widespread human suffering [19]. The Bengal Famine, aggravated by the fungal pathogen *Cochliobolus miyabeanus* inducing brown spot disease in rice [20], led to an estimated 2 to 3 million fatalities due to starvation and malnutrition [21,22]. Likewise, the Irish Potato Famine was instigated by *Phytophthora infestans*, the causative agent of late blight in potatoes [23]. *Phytophthora capsici*, an oomycete of great concern, induces root rot [24], stem blight, and fruit rot in various crops, notably peppers [25], particularly thriving in warm and moist environments [26]. The pathogen is disseminated through soil and water, utilizing zoospores that travel via soil water films and contaminated irrigation systems [27]. *Phytophthora capsici*, leading to significant economic repercussions amounting to millions of dollars annually, is especially evident in the United States [28].

The proteins 7VEM, 8H6Q, 8EBB, and 7XDS, derived from various devastating plant pathogens, represent critical targets for computational drug discovery aimed at combating agricultural diseases. The protein 7VEM, identified as NADPH-assisted quinone oxidoreductase from *Phytophthora capsici*, plays a crucial role in maintaining redox balance essential for the pathogen’s survival and virulence, making it a prime target for inhibitor design. Solved at 2.39 Å resolution with an R-work value of 0.167, its structural fidelity and lack of mutations ensure reliable insights for virtual screening and molecular docking. Similarly, the Class I sesquiterpene synthase BCBOT2 (apo) from *Botrytis cinerea* (PDB ID: 8H6Q), resolved at 2.00 Å with R-free and R-work values of 0.195 and 0.168 respectively, is vital for sesquiterpene biosynthesis linked to the pathogen’s virulence. Its structural data offer a robust framework for identifying small molecules to disrupt sesquiterpene synthesis, attenuating *B. cinerea*’s pathogenicity. The SIX6 protein from *Fusarium oxysporum* f. sp. *lycopersici*(PDB ID: 8EBB), despite its classification as a protein of unknown function, plays a critical role in the virulence mechanism of this vascular wilt pathogen, which devastates crops like tomatoes. Its high-resolution structure at 1.88 Å, with R-free and R-work values of 0.221 and 0.193, supports molecular docking, virtual screening, and lead optimization to design antifungal agents. Finally, the AvrSr35 effector protein from *Puccinia graminis*f. sp. *tritici*(PDB ID: 7XDS), resolved at 2.06 Å with reliable R-values (R-free: 0.287, R-work: 0.257, Observed: 0.258), provides critical insights into wheat stem rust pathogenicity. Its potential as a target for structure-based drug design is underscored by its well-defined active sites and suitability for molecular dynamics simulations and virtual screening. Together, these structural models offer a comprehensive platform for advancing the design of inhibitors to mitigate plant pathogen-induced diseases (Table 1).

**Table 1:** Protein information.

Title	PDB ID:7VEM	PDB ID:8H6Q	PDB ID:8EBB	PDB ID:7XDS
Organism	*Phytophthora capsici*	*Botrytis cinerei*	*Fusarium oxysporum*f. sp.*lycopersici*	*Puccinia graminis*f. sp.*tritici*
Resolution	2.39 Å	2.00 Å	1.88 Å	2.06 Å
R-Value Free	0.224	0.195	0.221	0.287
Ramachandran plot, %	89.5	93.8	91.8	94.7
References	[57]	[58]	[59]	[60]

*Botrytis cinerea*, a necrotrophic fungus [29], induces gray mold on more than 200 plant species, such as grapes, strawberries, and tomatoes [30], particularly thriving in environments characterized by high humidity and cool temperatures [31]. Its dissemination occurs through airborne conidia and entry into plants is facilitated through either wounds or natural openings [32–35]. The impact on yield can be substantial, with potential losses of up to 50% in crops affected by this pathogen [35–37], leading to significant economic loss estimated in the 10-100 billions of dollars globally on an annual basis [38–40]. *Fusarium oxysporum* f. sp. *lycopersici,* a soil-borne fungal pathogen [41–43], induces Fusarium wilt in tomatoes under favorable moist conditions [41,44]. Its dissemination occurs through contaminated soil, water, and plant matter, gaining access through roots and inhabiting the plant’s vascular system [45–47]. Severe infestations can lead to yield reductions in agricultural fields, with notable economic repercussions in tomato cultivation areas globally [48,49]. *Puccinia graminis* f. sp. *tritici*, a fungal pathogen accountable for stem rust in wheat [50], prospers under warm and moist conditions. Its dissemination occurs via airborne urediniospores capable of long-distance travel, infiltrating plants through stomata [51]. The potential yield reductions can be catastrophic, with losses escalating to 100% during severe outbreaks, significantly impacting worldwide wheat production and food security [52]. The rise of highly virulent strains underscoring the necessity for vigorous resistance breeding and comprehensive disease management tactics [53].The ongoing challenges presented by these fungal pathogens and the encouraging outcomes from computational investigations prompt this study to delve deeper into validating and exploring innovative targets for fungicidal activity through an integrated in silico methodology. Through the utilization of DFT, molecular docking, and MD simulations, the goal is to pinpoint and enhance potent inhibitors characterized by high specificity and minimal off-target effects, which could potentially facilitate the advancement of next-generation fungicides. This holistic approach holds the potential to enhance our comprehension of fungal pathogen mechanisms and make a significant contribution to the realms of sustainable agriculture and global food security.

## Methods

### Optimization and preparation of ligand

Material Studio 8.0 was used for extensive computational modeling and molecular optimization. The DMol3 algorithm was used for a thorough examination that included the identification of chemical descriptors, quantum characteristics, and geometric optimization. To provide precise and trustworthy findings, this procedure used the DFT functional in conjunction with the BLY3 basis set [54–56]. Utilizing the DFT functional, various quantum properties, includinglowest unoccupied molecular orbital(LUMO), highest occupied molecular orbital (HOMO), energy gap, ionization potential (I), electron affinity (A), chemical potential (μ), electronegativity (χ), hardness (η), softness (s), and electrophilicity (ω) were calculated using equations [5]:


$$E_{gap}=\left(E_{LUMO}–E_{HOMO}\right)$$ (1)

$$I=–E_{HOMO}$$ (2)

$$A=–E_{LUMO}$$ (3)

$$(X)\;=\;\frac{I+A}{2}$$ (4)

$$(\omega )=\frac{\mu^{2}}{2\eta }$$ (5)

$$\left(\mu \right)=-\frac{I+A}{2}$$ (6)

$$\left(\eta \right)=\frac{I-A}{2}$$ (7)

$$\left(S\right)=\frac{1}{\eta }$$ (8)

### Protein preparation and collection

The meticulous process of selecting and preparing the four fungal proteins, namely 7VEM (*Phytophthora capsica)*, 8H6Q (*Botrytis cinerea)*, 8EBB (*Fusarium oxysporum* f. sp*. lycopersici.)*, and 7XDS (*Puccinia graminis* f. sp. *tritici)*, was undertaken to ensure the reliability of the subsequent docking simulations. All of these proteins were found in different plant disease-causing fungi evaluated through the X-ray diffraction method with high stable configuration Ramachandran outliers listed (Table 1). These proteins were obtained from the protein data bank (PDB), which can be accessed at http://www.rcsb.org, on 5th August 2023. The selection of these proteins was based on their biological relevance, specifically their potential association with the study objectives. In order to create a clean protein template for docking using Discovery Studio, co-crystallized ligands, water molecules, and ions were removed. Additionally, the protein structures underwent energy minimization to optimize their conformations and alleviate any steric clashes. This ensured that the selected fungal proteins were properly prepared for accurate and meaningful molecular docking investigations.

### Molecular docking and visualization of docking

#### Molecular docking by PyRx.

The molecular docking analysis was conducted using PyRx software, employing the AutoDock Wizard option. To evaluate the binding affinity between the ligand and individual macromolecules, essential parameters (as detailed in Table 2) were obtained and utilized. In this process, the protein was loaded as a macromolecule, and the ligand was loaded separately. Subsequently, the loaded ligand underwent optimization for maximum energy, considering parameters such as grid surface area, center of grid, and grid dimensions. The goal was to ensure adequate coverage of the total surface area of both the ligand and the protein.The docking process was initiated with the specified parameters outlined (Table 2) within the PyRx software’s AutoDock Wizard option. Following the docking procedure, the resulting ligand-protein docking complex was further analyzed for non-covalent interactions using Discovery Studio visualization [61].

**Table 2:** Grid box parameters used for docking analysis in this study.

Protein Name with PDB ID	Grid Box Size	
	Center	Dimension(Å)
*Phytophthora capsici*(PDB: 7VEM)	X = −0.28	X = 83.52
	Y = −25.03	Y = 79.50
	Z = −7.46	Z = 72.44
*Botrytis cinerea* (PDB: 8H6Q)	X = 47.34	X = 95.68
	Y = 16.24	Y = 97.38
	Z = 19.13	Z = 80.33
*Fusarium oxysporum*f.sp. lycopersici(PDB: 8EBB)	X = 2.77	X = 70.97
	Y = 17.83	Y = 56.62
	Z = −11.09	Z = 67.38
*Puccinia graminis*f. sp. tritici(PDB: 7XDS)	X = 31.47	X = 63.80
	T = 75.30	Y = 78.47
	Z = 47.42	Z = 107.45

#### Molecular docking by Glide from Schrodinger suite.

In this research, glide docking was utilized as the molecular docking technique to examine the binding interactions between a variety of ligands and a target protein.The molecular docking was done using Glide tool on Schrodinger suite.For each protein structure, a grid box of 30 × 30 × 30 Å^3^ with a default inner box (20 × 20 × 20 Å^3^) was centered on the corresponding ligand [62]. There were however no boundaries and the setting parameters were set to default. Following that, conformational sampling was put into effect to the ligands, and their anticipated binding affinities were then utilized to evaluate them. The Schrödinger software suite includes the Glide docking tool, which enables a systematic search of the conformational space to facilitate a comprehensive examination of ligand-protein interactions. Considerations like attachment modes, bonding by hydrogen patterns, and energetics were thoughtfully taken into account when analyzing the docking results. This method produced insightful information about the connections between structure and activity as well as possible binding locations that are essential for the logical design of novel ligands with enhanced pharmacological profiles. By defining the binding (active) site residues, which were found, the binding site receptor grid for plant pathogenic fungal proteins was created. The docked conformers were evaluated using Glide (G) Score. The G Score is calculated as follows in equation [9]:


G  Score=a×vdW+b×coul+Lipo+HBond+Metal+BuryP+RotB+Site (9)

Wherein, vdW denotes van der Waals energy, Coul denotes Coulomb energy, Lipo denotes lipophilic contact, HBond indicates hydrogen-bonding, Metal indicates metal-binding, BuryP indicates penalty for buried polar groups, RotB indicates penalty for freezing rotatable bonds, Site denotes polar interactions in the active site and the a = 0.065 and b = 0.130 are coefficients of vdW and Coul.

### Pharmacokinetics and ADMET studies

The absorption, distribution, metabolism, excretion and toxicity is expressed in shortageby ADMET, which are valuable and necessary factors in the fungicide development process [63,64]. The ADMET criterion was obtained by use of the SwissADME and pkCSMonlinetool:http://biosig.unimelb.edu.au/pkcsm/prediction_single/adme_1643650057.59(accessed from the 10^th^October 2023) [65].

### Lipinski rule and pharmacokinetics

SwissADME, accessed on October 9, 2023, was utilized to predict pharmacokinetics and assess fungicide-likeness metrics. This online database, available at http://www.swissadme.ch/index.php [66], is respected for its important and adaptable functions that make information easier to access. In order to fully clarify the fungicide-like properties of different ligands, a large number of pharmacokinetic parameters were determined. These factors included the ligands’ lipophilicity, number of bond rotations (NRB), and molecular weight. The investigation also took into account the quantity of hydrogen bond acceptors (HBA) and donors (HBD). A thorough grasp of the ligands’ potential as fungicides was attained by closely analyzing these variables.

### Molecular dynamic

Simulations were conducted using the Desmond software suite (Schrödinger Release 2024-1: Desmond Molecular Dynamics System, D. E. Shaw Research, New York, NY, 2024) in accordance with established MD protocols. The molecular system, which consisted of a biologically relevant complex, was prepared using the OPLS-AA force field, and the solvent environment was described using an appropriate water model. To ensure solvent relaxation, the system underwent a staged equilibration process that involved restrained dynamics on the solute. Following this, production MD runs were carried out under NPT conditions, with a finite time step and temperature control. Desmond outputs trajectory files, energy logs, and other data that can be further analyzed or visualized using external tools. Trajectory analysis, performed using Maestro and supplemented by external tools, included measurements, such as root mean square deviation (RMSD), root mean square fluctuation (RMSF), radius of gyration, and energy profiles. Visualization tools were used to examine conformational changes and intermolecular interactions throughout the simulation [67]. This comprehensive computational investigation followed established best practices and parameters, providing a thorough exploration of the dynamic behavior of the molecular system under investigation.

## Results and discussion

### Optimized structure

**Fig 1:**
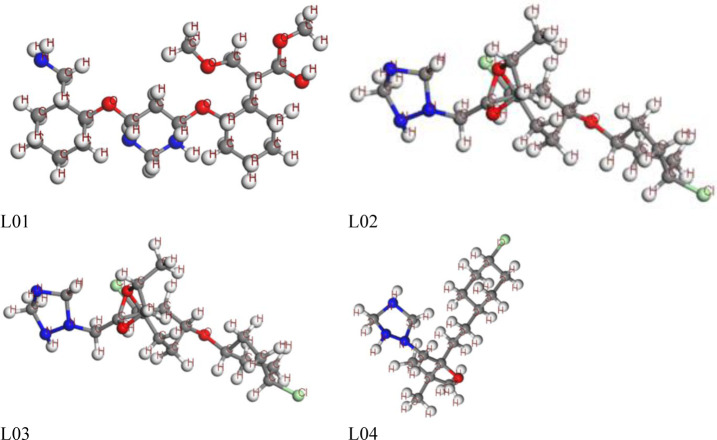
Optimized structure of inhibitors.

The study of a computational procedure to determine the quantum calculations of any chemical species requires the optimization of the molecular structure, which is an important aspect of its structural geometry [68]. Additionally, accurate computational parameters are obtained by determining the most stable configuration of any chemical structure. In this study, all compounds underwent computational optimization using the DFT functional, and their primary and most stable configuration was observed with minimal energy required for optimization. The antifungal ligands are Azoxystrobin(L01), Cyproconazole(L02), Difenoconazole (L03), Tebuconazole (L04), Tricyclazole (L05), Chlorothalonil (L06), Benalaxyl (L07),Bismerthiazol (L08), Carbendazim (L09), Hexaconazole (L10), Thiram (L11), Carboxin (L12), Iprodione (L13), Kresoxim-Methyl (L14), Cymoxanil (L15), Dichloran (L16), Propiconazole (L17), Dimethomorph (L18), Pyraclostrobin (L19) andAmetoctradin (L20) are shown (Figs 1 and S1).

### HOMO, LUMO, and chemical reactivity descriptors

The calculations of the molecular parameters, including LUMO, HOMO, energy gap (ΔE gap), chemical potential (µ), electronegativity (χ), hardness (η), softness (σ), and electrophilicity (ω), were conducted using the DFT functional, and the results are presented in Table 3. The HOMO-LUMO gap serves as a crucial indicator of chemical reactivity and stability in molecules. In this context, a wider gap suggests higher chemical stability, while a narrower gap implies increased reactivity [69–72]. The findings reveal that the HOMO–LUMO gaps range from 2.474 eV to 10.040 eV across all studied ligands. Notably, ligands L01 exhibit minor energy gaps and minimal softness values, indicating their potential for reactivity and reduced stability. In contrast, L14 stands out with the greatest hardness and the largest energy gap, emphasizing its stability.It is observed that the order of the energy gap is L01 < L10 < L13 < L15 < L18 < L19 < L07 < L02 < L09 < L20 < L03 < L05 < L06 < L17 < L08 < L12 < L04 < L14 < L11 < L16. (Table 3) further illustrates that the softness values are approximately 0.228 or less than 0.30, underscoring the potential for faster degradation and disintegration for elements with higher softness values [73–76]. Conversely, hardness, a crucial stability indicator, is reflected in the compounds’ resistance to changes in electron configuration [77–79]. Higher hardness values signify increased stability and resistance to changes, providing valuable insights into the chemical behavior of these compounds.

**Table 3:** Data of chemical descriptors.

Ligand	LUMO	HOMO	A = −LUMO	I = −HOMO	Energy gap = I−A	Chemical Potential (µ) = −I + A/2	Hardness (η) = I−A/2	Electronegativity (x) = I + A/2	Softness (σ) = 1/n	Electrophilicity (ω) = µ2/2η
L01	2.131	−9.534	−2.131	9.534	11.665	−3.701	5.832	3.701	0.171	1.174
L02	1.517	−9.034	−1.517	9.034	10.551	−3.758	5.275	3.758	0.189	1.338
L03	0.829	−8.111	−0.829	8.111	8.940	−3.641	4.470	3.641	0.223	1.482
L04	1.361	−9.076	−1.361	9.076	10.437	−3.857	5.218	3.857	0.191	1.425
L05	0.043	−8.943	−0.043	8.943	8.986	−4.450	4.493	4.450	0.222	2.203
L06	0.661	−9.386	−0.661	9.386	10.047	−4.362	5.023	4.362	0.199	1.894
L07	1.893	−8.727	−1.893	8.727	10.620	−3.417	5.310	3.417	0.188	1.099
L08	−1.074	−9.382	1.074	9.382	8.308	−5.228	4.154	5.228	0.240	3.289
L09	1.661	−9.441	−1.661	9.441	11.102	−3.890	5.551	3.890	0.180	1.363
L10	0.819	−8.843	−0.819	8.843	9.662	−4.012	4.831	4.012	0.207	1.665
L11	−2.417	−9.328	2.417	9.328	6.911	−5.872	3.455	5.872	0.289	4.990
L12	0.201	−8.810	−0.201	8.810	9.011	−4.304	4.505	4.304	0.221	2.056
L13	1.176	−9.643	−1.176	9.643	10.819	−4.233	5.409	4.233	0.184	1.656
L14	−1.003	−7.401	1.003	7.401	6.398	−4.202	3.199	4.202	0.312	2.759
L15	1.375	−9.528	−1.375	9.528	10.903	−4.076	5.451	4.076	0.183	1.524
L16	−2.474	−10.040	2.474	10.040	7.566	−6.257	3.783	6.257	0.264	5.174
L17	1.240	−8.880	−1.240	8.880	10.120	−3.820	5.060	3.820	0.197	1.441
L18	1.451	−9.976	−1.451	9.976	11.427	−4.262	5.713	4.262	0.175	1.590
L19	1.241	−9.036	−1.241	9.036	10.277	−3.897	5.138	3.897	0.194	1.478
L20	1.578	−9.137	−1.578	9.137	10.715	−3.779	5.357	3.779	0.186	1.333

### Frontier Molecular Orbital: HOMO and LUMO

**Fig 2:**
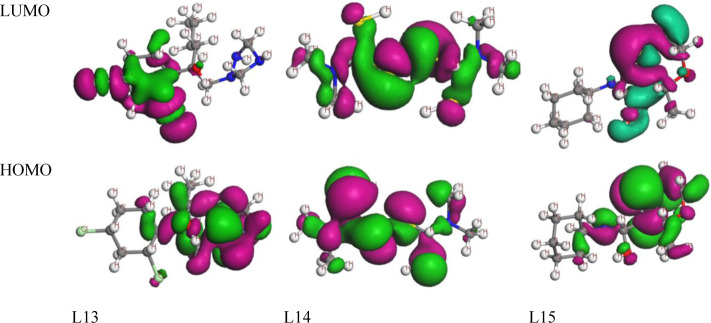
Frontier molecular orbitals diagram for HOMO and LUMO.

The frontier molecular orbital (FMO) was used to assess the kinetics, and the engaged regions where the protein could be folded become the active pharmacophore or active functional group. The maximum energy chemical orbital that an electron can occupy in a particular molecule is called the HOMO. Various chemical reactions include electrons in the HOMO, especially electron transfer processes. In addition to acting as the donor of electrons in reactions, the HOMO plays a critical role in nucleophilic assaults by contributing its electron density [80–84]. The lowest energy molecular orbital in a molecular structure that is free of electrons is known as the LUMO. During reactions, the LUMO and electrons from the HOMO of another molecule regularly interact. In electrophilic assaults, where it accepts electrons, the LUMO plays a crucial role as the electron acceptor during reactions. The dark blue color indicates the positive terminal of the orbitals in both LUMO, HOMO, while the pink color denotes the negative node. The more minor energy gap assists in the development of fungicide interaction with a protein. From the pictures in (Figs 2 and S2), there is various part of different molecules for HOMO and LUMO.

### Electrostatic potential map for Frontier Molecular Orbital

**Fig 3:**
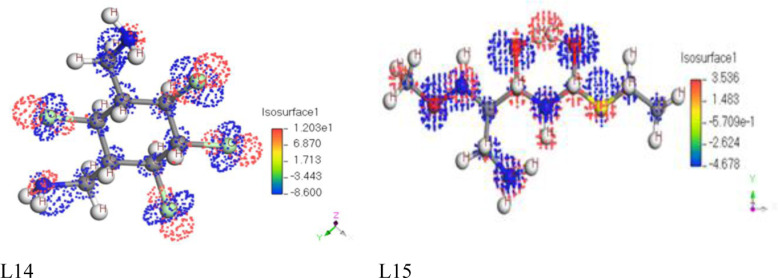
Electrostatic potential map for Frontier Molecular Orbital of L14 and L15.

Frontier Molecular Orbitals (FMOs) are a set of molecular orbitals that play a crucial role in the reactivity of a molecule. The two most important FMOs are the HOMO and the LUMO showed in the (Figs 3 and S3). These orbitals are involved in various chemical reactions and properties, especially in the context of organic chemistry. Electrostatic potential maps for the HOMO and LUMO can provide valuable insights into the reactivity and electronic structure of a molecule.

### Molecular docking

#### Auto docking by PyRx.

Molecular docking simulations were conducted to authenticate the pharmacological findings. These simulations confirmed the binding of fungicide compounds with the associated peptide in the presence of four-plant pathogenic fungal proteins, namely *Phytophthora capsica*, *Botrytis cinerea*, *Fusarium oxysporum* f. sp. *lycopersici*, and *Puccinia graminis* f. sp. *tritici*. The primary cause of binding between the compounds and proteins is the hydrogen and hydrophobic bonds, which are demonstrated by molecular docking. The interaction between protein and ligand is crucial for the development of structurally oriented fungicides. It is widely accepted that docking scores higher than -6.00 kcal/mol indicate a standard fungicide [85,86].Additionally, molecular docking is a reliable approach for understanding the engagement of two molecules and identifying the optimal configuration for ligand binding. Through *insilico* experiments, it was revealed that the fungicide compounds in Table 4 exhibit excellent binding affinity to the target proteins, with values ranging from −3.00 to −9.20 kcal/mol. Among the four fungal pathogens, the highest docking score of −8.20 kcal/mol was observed for *Phytophthora capsici*(7vem) in L03, while the lowest score of −3.80 kcal/mol was observed in L11. For *B. cinerei*(8H6Q), the highest docking score of −8.90 kcal/mol was found in L03, and the lowest score of −4.00 kcal/mol was found in L11. For *Fusarium oxysporum* f. sp. *lycopersici*(8EBB), the highest docking score of −8.40 kcal/mol was found in L03, and the lowest score of -3.80 kcal/mol was found in L11. Lastly, for *Puccinia graminis* f. sp. *tritici*(7XDS), the highest docking score of −9.20 kcal/mol was found in L18, and the lowest score of −4.30 kcal/mol was found in L11. These findings indicate that the fungicides (L01, L03, L04, L13, L14, L17, L18, and L19) exhibit significantly higher binding affinities against the four fungal pathogenic proteins compared to other ligands.

**Table 4:** Data of binding energy and name of interacted ligand against four fungal proteins in binding affinity (kcal/mol).

S.L	Compound	*Phytophthora capsica* (7VEM)	*B. cinerei*(8H6Q)	*Fusarium oxysporum*f. sp. *lycopersici* (8EBB)	*Puccinia graminis f.* sp. *Tritici*(7XDS)
L01	Azoxystrobin	−7.10	−8.20	−8.10	−7.60
L02	Cyproconazole	−6.80	−7.70	−6.60	−7.90
L03	Difenoconazole	−8.20	−8.90	−8.40	−7.80
L04	Tebuconazole	−6.80	−7.80	−7.20	−6.70
L05	Tricyclazole	−6.50	−7.40	−6.30	−7.00
L06	Chlorothalonil	−6.00	−6.20	−5.90	−5.90
L07	Benalaxyl	−6.50	−8.50	−6.90	−6.70
L08	Bismerthiazol	−6.10	−6.20	−6.20	−6.20
L09	Carbendazim	−6.30	−7.30	−6.40	−7.00
L10	Hexaconazole	−6.30	−7.40	−6.40	−6.60
L11	Thiram	−3.80	−4.00	−3.80	−4.30
L12	Carboxin	−6.60	−7.20	−6.10	−7.20
L13	Iprodione	−8.10	−8.10	−6.80	−7.90
L14	Kresoxim-Methyl	−6.60	−8.50	−7.00	−8.30
L15	Cymoxanil	−5.60	−6.10	−5.60	−6.10
L16	Dichloran	−5.30	−5.90	−5.50	−5.90
L17	Propiconazole	−7.20	−7.90	−7.20	−8.40
L18	Dimethomorph	−7.60	−8.20	−7.60	−9.20
L19	Pyraclostrobin	−8.00	−8.40	−8.10	−8.70
L20	Ametoctradin	−6.20	−7.30	−5.50	−6.50

### Molecular docking by Glide from Schrodinger suite

Schrodinger suite ligand preparation product, Ligprep was used to prepare high quality, all atom 2D structures. The ligand preparation included 2D–3D conversions, generating variations, correction, verification and optimization of the structures. Receptor grid was generated using Receptor grid generation in the Glide application of Maestro (Schrödinger Release 2024-1: Glide, Schrödinger, LLC, New York, NY, 2024). Through in silico experiments, it was revealed that the fungicide compounds in Table 5 exhibit excellent binding affinity to the target proteins, with values ranging from −2.692 to −7.317 kcal/mol. Among the four fungal pathogens, the highest docking score of −7.317 kcal/mol was observed for *Phytophthora capsica* (7vem) in L03, while the lowest score of −3.184 kcal/mol was observed in L20. For *B. cinerei*(8H6Q), the highest docking score of −7.270 kcal/mol was found inL09, and the lowest score of −3.317 kcal/mol was found in L15.For *Fusarium oxysporum* f. sp. *lycopersici* (8EBB), the highest docking score of −6.418 kcal/mol was found in L07, and the lowest score of −3.800 kcal/mol was found in L11. Lastly, for *Puccinia graminis* f. sp. *tritici*(7XDS), the highest docking score of −5.737 kcal/mol was found in L04, and the lowest score of −2.448 kcal/mol was found in L14.

**Table 5:** Data of binding energy and name of interacted ligand against 4 plant fungal pathogens in binding affinity (kcal/mol).

S.L	Compound	*Phytophthora capsici*(7vem)	*Botrytis cinerea*(8H6Q)	*Fusarium oxysporum*f.sp*. Lycopersici*(8EBB)	*Puccinia graminis*f.sp. *tritici*(7XDS)
L01	Azoxystrobin	−7.044	−4.631	−4.631	−5.392
L02	Cyproconazole	−6.074	−6.109	−5.487	−5.317
L03	Difenoconazole	−7.317	−6.499	−5.779	−5.335
L04	Tebuconazole	−7.061	−6.731	−5.184	−5.737
L05	Tricyclazole	−5.633	−5.707	−5.648	−4.332
L06	Chlorothalonil	−5.137	−5.603	−5.065	−3.899
L07	Benalaxyl	−5.457	−4.494	−6.418	−2.692
L08	Bismerthiazol	−4.294	−3.781	−3.786	−3.729
L09	Carbendazim	−5.582	−7.270	−6.042	−4.442
L10	Hexaconazole	−5.555	−6.498	−5.712	−3.456
L11	Thiram	−4.276	−5.294	−4.099	−3.065
L12	Carboxin	−5.760	−6.024	−4.946	−2.933
L13	Iprodione	−5.580	−5.331	−5.490	−3.893
L14	Kresoxim-Methyl	−5.132	−4.403	−4.896	−2.448
L15	Cymoxanil	−3.976	−3.830	−3.317	−3.501
L16	Dichloran	−5.585	−5.675	−5.370	−4.097
L17	Propiconazole	−6.068	−5.624	−5.468	−3.701
L18	Dimethomorph	−6.068	−5.624	−5.468	−5.285
L19	Pyraclostrobin	−6.310	−5.697	−5.719	−3.701
L20	Ametoctradin	−3.184	−4.232	−4.123	−3.828

### A comparative study for docking results

**Fig 4:**
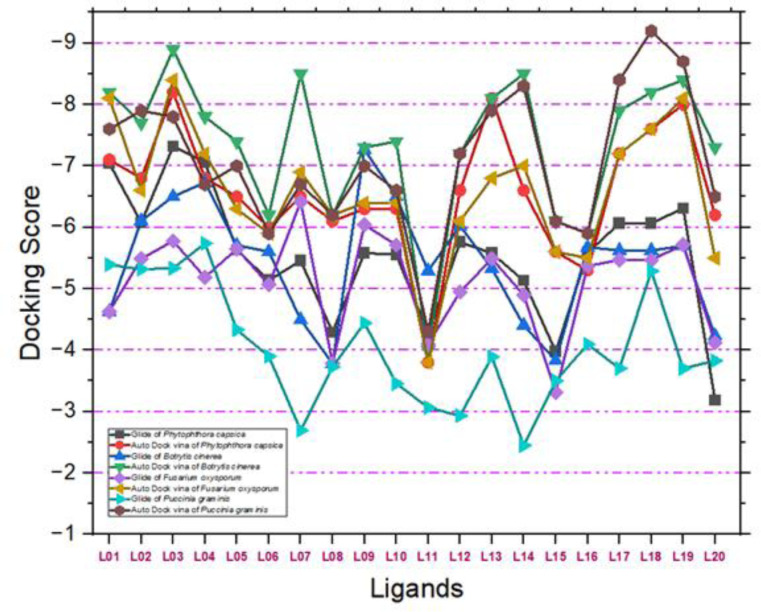
A comparative study of AutoDock by PyRx and Molecular Docking by Glide by Schrödinger suite.

The comparative analysis of docking results obtained from AutoDock by PyRx and Molecular Docking by Glide from the Schrödinger suite underscores the robust binding affinities of certain fungicide compounds with plant pathogenic fungal proteins. In (Fig 4), AutoDock yielded docking scores ranging from −3.00 to −9.20 kcal/mol, with the highest affinity observed for *Phytophthora capsici*(7vem) with Ligand L03 at −8.20 kcal/mol. Glide results corroborated this finding, producing scores from −2.692 to −7.317 kcal/mol and identifying ligand L03 as having the highest affinity for *Phytophthora capsica* with a score of −7.317 kcal/mol. Notably, both methods consistently identified L03 as having the strongest binding affinity for this pathogen, highlighting its potential efficacy.

Considering the biological systems in contact with these molecules, ligand L03’s high affinity suggests it could effectively inhibit the growth and proliferation of *Phytophthora capsici*, a significant plant pathogen. The interaction of L03 with the fungal proteins disrupt critical biological processes within the fungus, thereby mitigating infection and disease spread in plants. However, it is crucial to evaluate the potential impact of L03 on non-target organisms, including beneficial soil microbes, other plant species, and possible environmental persistence. The favorable binding scores indicate that L03 can be developed as an effective fungicide, but comprehensive studies on its environmental behavior, toxicity to non-target species, and overall safety are essential to ensure sustainable and safe agricultural practices. This holistic approach will help in leveraging L03’s potential while minimizing adverse ecological impacts, thereby contributing to effective integrated pest management strategies.

### Protein-Ligand interaction for auto docking

**Fig 5:**
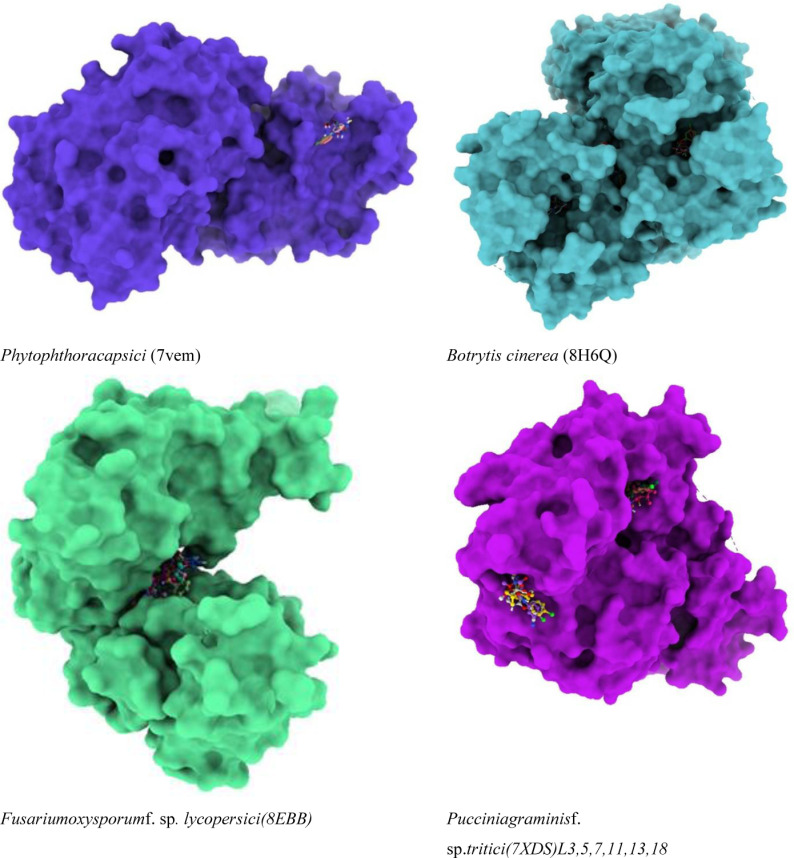
Molecular docking poses of four plant fungal pathogen proteins with 20 anti-fungal ligands.

The most important factor to take into account when determining which fungicide is most effective is the ligand-protein interaction, which is achieved through the formation of weak bonds or covalent bonds. This interaction provides an approximation of the binding affinity or energy of substances with the proteins of micro pathogens where the molecular docking poses of four plant fungal pathogen proteins with 20 anti-fungal ligands mentioned in (Fig 5). The bond distance was assessed to have a better understanding of the interaction between the molecule and the protein associated with the chosen fungal infections. Substitute data indicates that there are various types of bonds, including hydrophilic, hydrophobic, Van der Waal, and H-bonds. Furthermore, the protein’s locations that the ligand binds are identified. According to the results, the ligand L03 has the most binding sides, with an H-bond count of five and six hydrophobic bonds against the *B. cinerei*- 8H6Q protein.

### Different poses of docking for protein ligand interaction

#### PyRx Docking.

**Fig 6:**
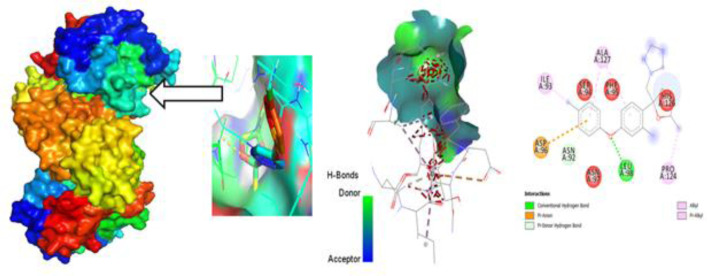
Molecular docking poses of plant pathogenic fungal protein *Phytophthoracapsici* (PDB: 7vem) with L03.

**Fig 7:**
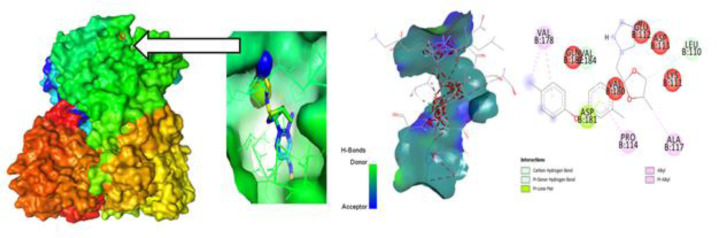
Molecular docking poses of plant pathogenic fungal protein *Botrytis cinerea (*PDB: 8H6Q) with L03.

**Fig 8:**
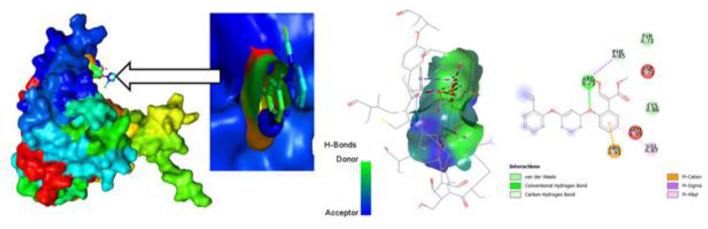
Molecular docking poses of plant pathogenic fungal protein *Fusarium oxysporum* f. sp. Lycopersici(PDB: 8EBB) withL01.

**Fig 9:**
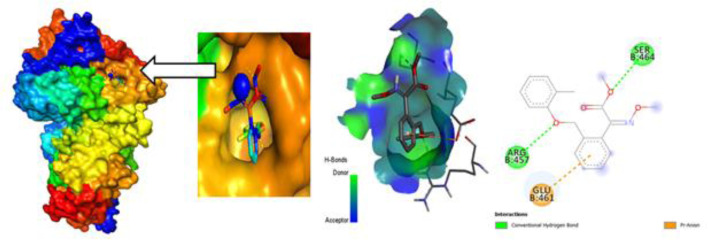
Molecular docking poses of plant pathogenic fungal protein *Pucciniagraminis* f. sp. *tritici (*PDB: 7XDS) with L18.

The ligand binding sites with receptor was identified with the help of Discovery Studio version 2020, and graphically represents (Figs 6–9) as well as (S4 Fig). In this case, at first, auto docking has been performed on the protein and ligand to identify the binding sites and obstructing the active site, as well as determining the amino acid residue. The mostly present bond is hydrogen and hydrophobic bond, and they are responsible for docking score variation. After performing molecular docking to predict the interaction between a drug and a protein pocket, several post-docking steps and analyses are typically carried out to understand and evaluate the results. If available, docking results are frequently compared to experimental data to verify the precision of the predictions. A comparison of anticipated binding modes with crystallographic or other experimental structures may be used for validation. To make sure it predicts known binding affinities appropriately, the scoring function utilized during docking is assessed. Future projections may be more accurate if relevant improvements are made. So, what, all drugs are in the molecular pocket of proteins, so the docking procedure, several post-docking steps and analyses are valid.

### Protein ligand interaction from Glide docking

**Fig 10:**
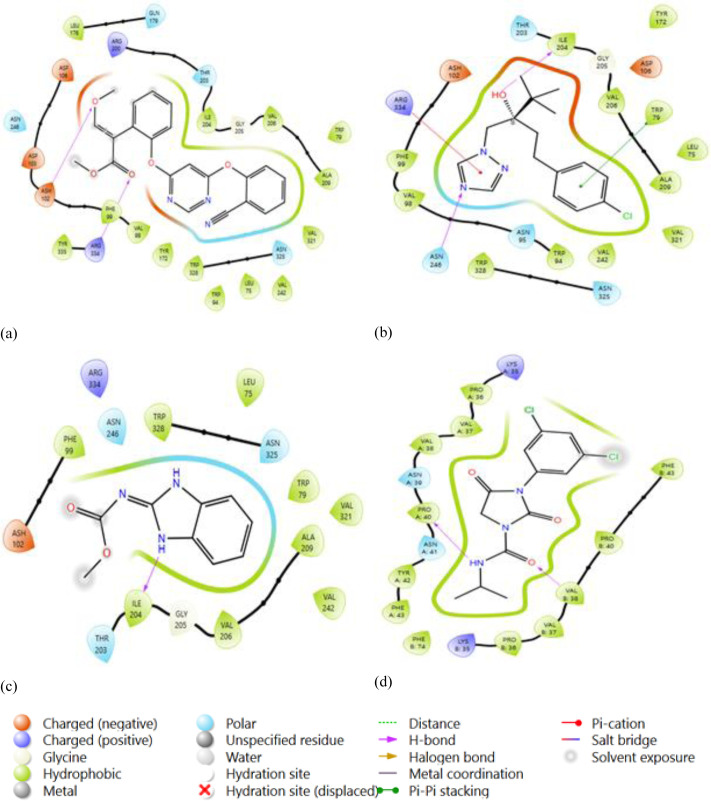
2D stick diagram of (A) Azoxystrobin; (B) Tebuconazole; (C) Carbendazim; (D) Iprodione; illustrating hydrogen bonds and pi-pi stacking formed with the amino-acid residues at the binding pocket of Botrytis cinerea (8H6Q).

Glide, developed by Schrödinger, is a widely used molecular docking program that predicts the binding mode and affinity of a small molecule (ligand) to a target protein. Analyzing protein-ligand interactions from Glide docking involves examining the docking results and visualizing the binding mode exhibited (Fig 10). Consider the docking score provided by Glide as an estimate of the binding affinity. Lower scores generally indicate better binding.

After performing molecular docking to predict the interaction between a drug and a protein pocket, several post-docking steps and analyses are typically carried out to understand and evaluate the results. Numerous binding poses, or hypothesized binding configurations, have been generated for the drug by the docking software inside the protein pocket. A score has been allocated to each pose, which symbolizes the interaction’s expected binding energy or inclination. On average, lower scores indicate more favorable binding. To make sure it predicts known binding affinities appropriately, the scoring function utilized during docking is assessed. Future projections may be more accurate if relevant improvements are made.

### Pharmacokinetics and fungicide-likeness study

Pharmacological fungicide-likeness is a groundbreaking evaluation of the potential of a particular chemical used as an oral medicine with respect to bioavailability. It is estimate that nine out of twelve targeted medicines are not transparently changed due to their negative effect, resulting in significant medication costs, time, and human resources being wasted [87]. This problem occurs due to failure to identify the actual fungicide characteristics. However, by employing a new approach, Lipinski’s five-rule, it is possible to readily test the aspects of lead compounds, such as their bioavailability and G.I. absorption, among other things [88].

From Table 6 it can be seen that all the ligands follow the Lipinski’s five-rule so it can be said that they can remain active as potential drug as small molecules. Each drug can display 0.55 bioavailability score, and they do not violate any conditions of Lipinski’s five-rule.

**Table 6:** Data of Lipinski rule, pharmacokinetics, and fungicide likeness.

Ligand No	Compound	Molecular weight, g/mol	Number of Rotatable bonds	Hydrogen Bond acceptor	Hydrogen Bond donor	Topological polar surface area (Å²)	Lipinski rule		Bioavailability score
							result	violation	
L01	Azoxystrobin	403.39	8	8	2	103.56	Yes	0	0.56
L02	Cyproconazole	291.78	5	3	0	50.94	Yes	0	0.55
L03	Difenoconazole	406.26	5	5	1	58.40	Yes	0	0.55
L04	Tebuconazole	307.82	6	3	0	50.94	Yes	0	0.55
L05	Tricyclazole	189.24	0	2	1	58.43	Yes	0	0.55
L06	Chlorothalonil	265.91	0	2	1	47.58	Yes	0	0.55
L07	Benalaxyl	325.40	7	3	0	46.61	Yes	0	0.55
L08	Bismerthiazol	278.40	4	2	2	202.08	Yes	0	0.55
L09	Carbendazim	191.19	3	3	1	67.01	Yes	0	0.55
L010	Hexaconazole	314.21	6	3	0	50.94	Yes	0	0.55
L011	Thiram	240.43	5	0	0	121.26	Yes	0	0.55
L012	Carboxin	235.30	3	2	0	63.63	Yes	0	0.85
L013	Iprodione	330.17	4	3	1	69.72	Yes	0	0.55
L014	Kresoxim-Methyl	313.35	7	5	0	57.12	Yes	0	0.55
L015	Cymoxanil	198.18	6	5	1	103.58	Yes	0	0.55
L016	Dichloran	207.01	1	2	1	71.84	Yes	0	0.55
L017	Propiconazole	342.22	5	4	1	49.17	Yes	0	0.55
L018	Dimethomorph	387.86	6	4	0	48.00	Yes	0	0.55
L019	Pyraclostrobin	387.82	8	5	0	65.82	Yes	0	0.55
L20	Ametoctradin	275.39	8	3	0	69.10	Yes	0	0.55

### Bio-availability and toxicity of the fungicides

The bioavailability and toxicity profiles of the twenty ligands reveal significant variations in their absorption, permeability, blood-brain barrier (BBB) penetration, and interactions with P-glycoprotein, cation transporters, and CYP450 enzymes. Most ligands exhibit high absorption in the human intestine, with L02, L03, L04, L05, L09, L12, L13, L14, L15, and L20 approaching or achieving near-complete absorption added in Table 7. Ligand L05 shows excellent BBB penetration, indicating potential central nervous system activity, whereas L01 and L02 exhibit moderate permeability. The interaction with P-glycoprotein varies, with L11 and L12 showing significant inhibition, which could influence drug-drug interactions and efflux. Additionally, the renal organic cation transporter interactions highlight L11 and L15 as noteworthy due to their high scores. Most ligands are substrates for CYP450 enzymes, with L01, L04, L05, L10, L14, L15, L17, and L18 showing notable inhibitory activity, suggesting potential for metabolic interactions. The sub-cellular localization data further suggest diverse intracellular distribution, with L01, L05, L07, L08, L10, L12, and L20 showing significant localization, possibly indicating targeted intracellular effects. Overall, L11 and L12 emerge as particularly potent candidates given their comprehensive favorable profiles across multiple parameters, while others like L01 and L09 demonstrate lower permeability and reactivity, potentially impacting their efficacy and toxicity profiles.

**Table 7:** Bio-availability and toxicity of the fungicides.

Ligand	Absorption Human Intestinal	Caco-2 Permeability	Blood-Brain Barrier (BBB)	P- I glycoprotein inhibitor	Cation Transporter Renal Organic	P- II glycoprotein substrate	Sub-cellular localization	Substrate CYP450 2C9	Inhibitor CYP450 1A2
L01	0.5107	0.5663	0.6630	0.7237	0.6170	0.5442	0.8384	0.8069	0.7507
L02	0.9812	0.5617	0.7017	0.6006	0.7102	0.8528	0.5908	0.7075	0.7079
L03	0.9674	0.5825	0.6071	0.7615	0.6265	0.8671	0.4655	0.8009	0.6706
L04	0.9745	0.5742	0.5549	0.8515	0.6636	0.8712	0.6484	0.7393	0.7171
L05	0.9915	0.5075	0.9372	0.5704	0.5913	0.6617	0.5332	0.8212	0.6994
L06	0.8851	0.5476	0.9355	0.6096	0.7651	0.7811	0.5515	0.7726	0.5323
L07	0.8963	0.6421	0.9567	0.8274	0.7913	0.7518	0.5677	0.7434	0.7794
L08	0.6986	0.5799	0.9532	0.5613	0.7639	0.7321	0.5078	0.8535	0.8911
L09	0.9861	0.5288	0.9454	0.7666	0.6042	0.6150	0.6030	0.7474	0.5490
L10	0.9724	0.5886	0.5579	0.6053	0.5000	0.9115	0.4643	0.6683	0.6965
L11	0.9625	0.5000	0.9664	0.9950	0.9064	0.8586	0.4894	0.8228	0.5176
L12	0.9960	0.5708	0.9606	0.9479	0.7373	0.6943	0.6446	0.6602	0.5136
L13	0.0992	0.6004	0.6893	0.9696	0.8378	0.7551	0.6052	0.7278	0.6798
L14	0.9930	0.5480	0.9247	0.7869	0.6053	0.6295	0.7294	0.7948	0.8582
L15	0.9971	0.6077	0.9144	0.7309	0.9168	0.6570	0.8082	0.8151	0.739
L16	0.5153	0.5753	0.7250	0.7997	0.7941	0.5927	0.4554	0.7457	0.5622
L17	0.7399	0.5911	0.6208	0.9683	0.6607	0.8207	0.4652	0.8073	0.6451
L18	0.9758	0.5269	0.7663	0.8813	0.5153	0.8390	0.5495	0.8533	0.7283
L19	0.9799	0.6043	0.6700	0.6214	0.7787	0.5211	0.6468	0.6860	0.6461
L20	1.0000	0.5205	0.9108	0.6888	0.5000	0.5886	0.3373	0.8896	0.5000

### Molecular dynamics

#### Root Mean Square Deviation (RMSD).

For exploring the structural inflexibility and validating the docking scenarios of the topmost ligand-protein complexes *Phytophthora capsici*(7vem) – Difenoconazole, *Botrytis cinerea* (8H6Q) – Carbendazim, *Fusarium oxysporum* f. sp*. Lycopersici* (8EBB) - Difenoconazole, *Puccinia graminis* f. sp*. triti*ci (7XDS), MD simulations were performed for 100 ns. By analyzing the C-alpha atom’s RMSD, the firmness of the ligand-protein complexes was assessed.

**Fig 11:**
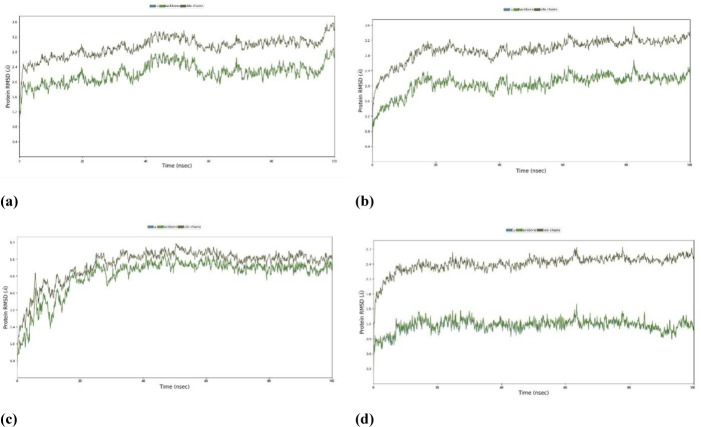
Root Mean Square Deviation (RMSD) of protein-ligand complexes. complexes (a) Phytophthoracapsici (7vem) – Difenoconazole, (b) Botrytis cinerea (8H6Q) – Carbendazim, (c) Fusariumoxysporum f. sp. Lycopersici (8EBB) –Difenoconazole, (d) Pucciniagraminis f. sp. tritici (7XDS).

Protein complexes exhibit initial fluctuations, followed by a transition to a stable state. When the RMSD values fall below 1.8 Å (Angstroms), it typically suggests a robust structural alignment or similarity between the compared structures (Fig 11). An RMSD below 1.8 Å signifies a significant degree of structural similarity, indicating that the examined structures closely resemble each other in terms of both overall form and atomic positions. Attaining RMSD values below 1.8 Å is commonly regarded as an indication of the precision of predicted structures in simulation and molecular modeling studies. This implies a close resemblance between the modeled structure and the reference or experimental structure.

#### Root Mean Square Fluctuation (RMSF) with respect of residues.

The RMSF with respect to residues is a measure commonly used in the analysis of MD simulations or experimental data of proteins or other biomolecules. It provides insights into the flexibility or mobility of individual amino acid residues within a protein structure. RMSF is calculated as the square root of the average of the squared displacements (or fluctuations) of each residue from its average position over the course of a simulation or experiment. Mathematically, for a protein with N residues, the RMSF of residue i (RMSF and RMSFi) is calculated as:


$$RMSFi=\sqrt{\frac{1}{n}}\sum_{j=1}^{n}{\left(x_{ij\;}-{\bar{x}}_{i}\right)}^{2}$$

Where, *n* is the number of frames or snapshots in the simulation or experimental data, *xij* is the position of residue, *i* in frame j, and *xˉi* is the average position of residue i over all frames.

**Fig 12:**
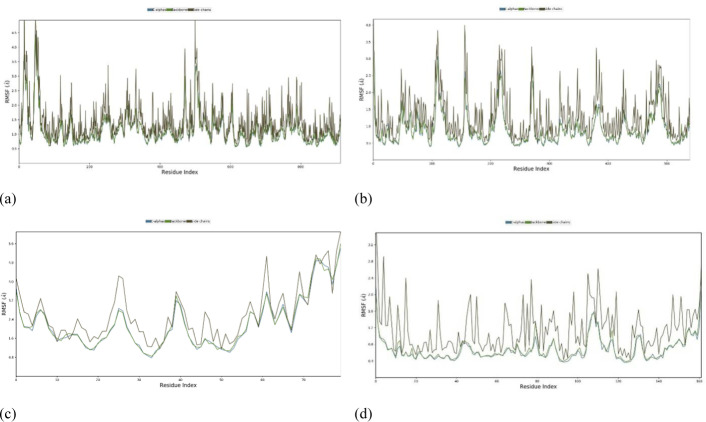
Root Mean Square Fluctuation with respect of residuesprotein-ligand complexes. (a) *Phytophthora capsici* (7vem) – Difenoconazole, (b) *Botrytis cinerea* (8H6Q) – Carbendazim, (c) *Fusariumoxysporum* f. sp. *Lycopersici* (8EBB) –Difenoconazole, (d) *Pucciniagraminis* f. sp. *tritic*i (7XDS).

In addition, the high RMSF values for certain residues indicate that those residues are more flexible or dynamic. Low RMSF values suggest that those residues are more rigid or less dynamic. RMSF values can be correlated with structural features, functional regions, or interactions within the protein. For example, loops and flexible regions tend to have higher RMSF values compared to core secondary structure elements such as alpha helices and beta strands. It is seen (Fig 12) that the RMSF shows the much less value mean that they are not only excess the value of 2.5 or more. So that it is said that the docked configuration more stable and may be acted as drug after docking accounting by docking. However, it is a valid procedure.

#### Protein secondary structure elements (SSE).

**Fig 13:**
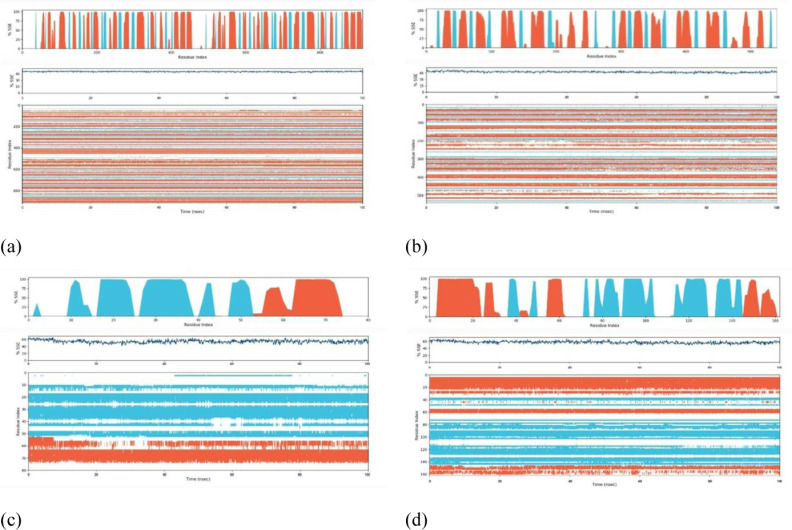
Protein secondary structure elements (SSE) of protein- ligand complexes. (a) *Phytophthoracapsici* (7vem) – Difenoconazole, (b) *Botrytis cinerea* (8H6Q)–Carbendazim, (c) *Fusariumoxysporum* f. sp. *Lycopersici* (8EBB) –Difenoconazole, (d) *Pucciniagraminis* f. sp. *tritici* (7XDS).

Protein secondary structure elements (SSEs) are recurring patterns in protein structures that arise from hydrogen bonding interactions between amino acid residues shown (Fig 13). Common SSEs include alpha helices, beta strands, and loops or turns. In MD analysis, identifying and analyzing SSEs can provide insights into the dynamic behavior and stability of proteins. SSEs can be identified using various algorithms or methods. Commonly used algorithms include DSSP (Define Secondary Structure of Proteins), STRIDE (Structural Identification), and VMD (Visual Molecular Dynamics) among others. These algorithms assign secondary structure elements to each residue in a protein based on the local hydrogen bonding patterns and dihedral angles. Alpha helices are characterized by a regular pattern of hydrogen bonds between residues, resulting in a helical structure. Beta strands form when adjacent segments of the polypeptide chain align in an extended conformation and form hydrogen bonds with each other. Loops or turns are regions of the protein structure that do not form regular hydrogen bonding patterns and connect alpha helices and beta strands. Once SSEs are identified, various analyses can be performed to understand their dynamics and interactions. Quantitative measures such as the length of alpha helices or beta strands, the number of hydrogen bonds within SSEs, and the persistence of SSEs over time can be calculated. Changes in SSEs during the simulation, such as the formation or disruption of helices or strands, can be monitored to study protein folding, unfolding, or conformational changes. SSEs can also be correlated with other structural or dynamic properties, such as solvent accessibility, protein-ligand interactions, or protein-protein interactions. However, SSEs, and SASA mention the stable configuration of docked complexes.

#### Protein Ligand interaction by bond.

In MD simulations, the interactions between a protein and a ligand (small molecule) are crucial for understanding the mechanism of binding and the stability of the complex. These interactions can involve various types of bonds, including covalent bonds, hydrogen bonds, hydrophobic interactions, and electrostatic interactions. Covalent bonds between the protein and ligand can form when specific reactive groups are present.

**Fig 14:**
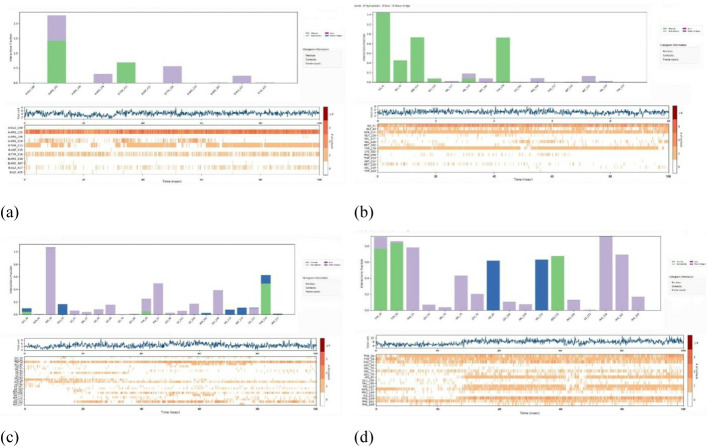
Protein Ligand interaction by bond of protein- ligand complexes. (a) *Phytophthoracapsici* (7vem) – Difenoconazole, (b) *Botrytis cinerea* (8H6Q)–Carbendazim, (c) *Fusariumoxysporum* f. sp. Lycopersici (8EBB) –Difenoconazole, (d) *Pucciniagraminis* f. sp. *tritici* (7XDS).

Analysis involves identifying the formation or breaking of covalent bonds over the course of the simulation. These interactions are typically less common in non-covalent protein-ligand interactions but are important in certain types of enzyme-substrate interactions or covalent inhibitors. Hydrogen bonds between the protein and ligand play a significant role in stabilizing the complex. Analysis involves identifying hydrogen bond formation and rupture events. Criteria such as distance and angle cutoffs are used to define hydrogen bonds. The frequency, duration, and strength of hydrogen bonds can be analyzed to understand their contribution to binding affinity attached (Fig 14). Hydrophobic interactions occur between non-polar regions of the ligand and protein. Analysis involves identifying hydrophobic contacts based on the proximity of non-polar atoms. Solvent-accessible surface area (SASA) analysis can also provide insights into the burial of hydrophobic residues upon ligand binding.

#### Radius of gyration (Rg) of WT, mutations, Solvent-accessible surface area (SASA).

The distribution of an object’s mass around its axis of rotation is measured by the radius of gyration, or Rg. It is frequently used to characterize the spatial distribution of mass in a rigid body or a system of particles in physics and engineering. The radius of gyration for a particular system or object is equal to the square root of the moment of inertia divided by the total mass. In terms of math, it is stated as:


$$\mathrm{Rg}=\sqrt{\frac{I}{\text{m}}}$$

Here,

Rg is the radius of gyration, I is the moment of inertia of the object or system,m is the total mass.

**Fig 15:**
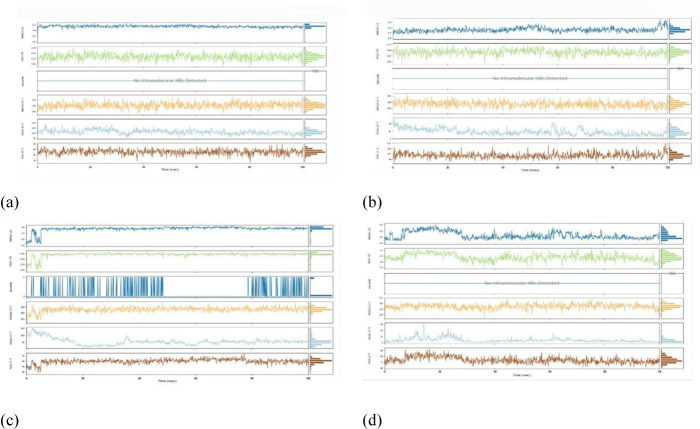
Radius of gyration (Rg) of WT, mutations, Solvent-accessible surface area (SASA). (a) *Phytophthoracapsici* (7vem) – Difenoconazole, (b) *Botrytis cinerea* (8H6Q)–Carbendazim, (c) *Fusariumoxysporum* f. sp. Lycopersici (8EBB) –Difenoconazole, (d) *Pucciniagraminis* f. sp. *tritici* (7XDS).

From the radius of gyration data, it is found that stability and response to external forces under different loading conditions of structure is valid and stable. In MD simulations, the solvent-accessible surface area (SASA) is an essential metric that offers important details on how atoms in a bimolecular system are exposed to the surrounding solvent. SASA is frequently used to investigate how ligands and proteins interact. Variations in the solvent accessibility can reveal areas that are revealed during unbinding or concealed during ligand binding. Finding binding locations and comprehending the energetics of protein-ligand interactions benefit from this. From data of MD simulations, with SASA analysis, it helps to show that the working procedure of docking is valid by experimental data by providing insights into the dynamic behavior of molecules, revealing solvent exposure and flexibility that may not be evident in static structures shown (Fig 15).

#### Torsional flexibility in Molecular Docking studies.

Ligands frequently have chemical structures that are flexible, and their torsional flexibility—the capacity to rotate around particular bonds—allows them to take on many conformations. In order for the ligand to locate an energetically advantageous binding position within the protein’s binding site, this flexibility is essential. Torsional flexibility is taken into account by the software while simulating the interaction between a ligand and a protein. In order to investigate several conformations and determine the ideal binding posture inside the protein’s binding site, the ligand is permitted to rotate around its rotatable links.

In MD simulations of protein-ligand interactions, torsion refers to the rotation of specific dihedral angles around covalent bonds within the ligand or the protein. Molecular dynamics simulations involve the computational modeling of the dynamic behavior of molecules over time. Torsional movements are crucial in understanding the conformational changes and flexibility of both the protein and the ligand during their interaction.

**Fig 16:**
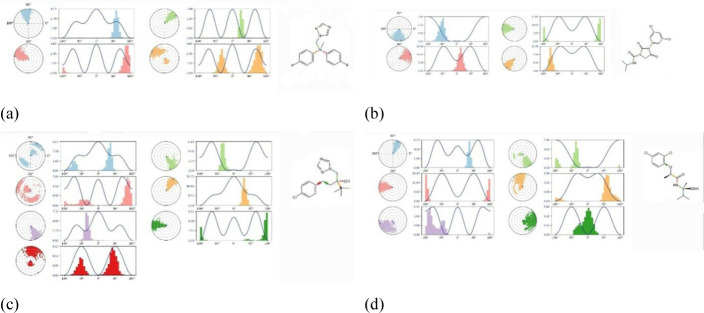
Torsional flexibility In Molecular Docking Studies protein- ligand complexes. protein-ligand complexes (a) *Phytophthoracapsici* (7vem) – Difenoconazole, (b) *Botrytis cinerea* (8H6Q)–Carbendazim, (c) *Fusariumoxysporum* f. sp. *Lycopersici* (8EBB) –Difenoconazole, (d) *Pucciniagraminis* f. sp. *tritici* (7XDS).

First of all, MD simulations track the positions and movements of atoms over time, allowing for the observation of conformational changes. Torsional movements influence the overall flexibility and shape of the protein-ligand complex from the first picture where most of residues are in right shape among 90−180. Secondly, the torsional energy landscape provides insights into the stability and energetically favorable conformations of the protein-ligand complex. All of residues are in lower energy almost zero energy, showing the highest stability having (Fig 16).

### Ramachandran plot for Docked protein complex

**Fig 17:**
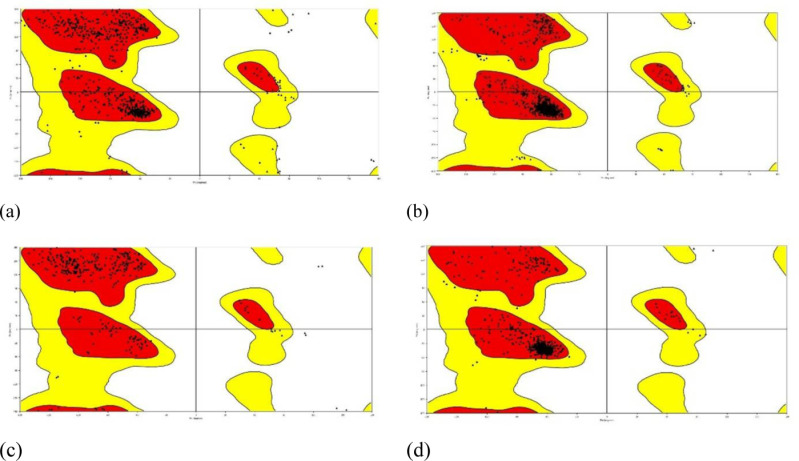
Torsional flexibility In Molecular Docking Studies protein- ligand complexes. (a) *Phytophthoracapsici* (7vem) – Difenoconazole, (b) *Botrytis cinerea* (8H6Q)–Carbendazim, (c) *Fusariumoxysporum* f. sp. Lycopersici (8EBB) –Difenoconazole, (d) *Pucciniagraminis* f. sp. *tritici* (7XDS).

The Ramachandran plot’s precise characteristics and importance would be described in the pertinent literature or documentation if it is, in fact, a tool or analysis approach used to evaluate the stability of proteins. Studying a variety of elements, including protein folding, thermodynamic characteristics, and interactions with other molecules, is frequently necessary for the stability analysis of proteins. The statistical distribution of the possible combinations of the backbone the dihedral angles υ and ψ is presented in the Ramachandran the following diagram. The Ramachandran plot’s permissible regions, in theory, indicate the potential values of the Phi/Psi angles for an amino acid (X) in an ala-X-ala tripeptide. It can be seen that the plots generally favor amino acid resets above 96%, which give the protein ligand complex a double configuration taken from (Fig 17).

## Conclusion

The combination of in silico investigations using AutoDock by PyRx and Molecular Docking by Glide from the Schrödinger suite has yielded valuable insights into potential fungicide candidates against four plant pathogenic fungal proteins -*Phytophthora capsici, Botrytis cinerea, Fusarium oxysporum* f. sp. *lycopersici,* and *Puccinia graminis* f. sp. *tritici*. The robust molecular docking simulations have convincingly validated the pharmacological findings, demonstrating the binding of various fungicide compounds to their respective target proteins. The main modes of interaction between the compounds and proteins, revealed through these simulations, mostly involve hydrogen and hydrophobic bonds. These interactions play a crucial role in the development of structurally-oriented fungicides, highlighting the importance of understanding the intricate engagement between proteins and ligands. The accepted standard for a fungicide, with docking scores greater than −6.00 kcal/mol, aligns with the results, demonstrating the potential effectiveness of the identified compounds. Among the four fungal pathogens, the fungicides L01, L03, L04, L13, L14, L17, L18, and L19 consistently exhibited significantly higher binding affinities compared to other ligands, with docking scores ranging from −3.00 to −9.20 kcal/mol. In case of DFT, L16 demonstrates the highest electrophilicity (5.174), indicating its strong potential as an electron acceptor, with high chemical stability due to its significant hardness (3.783). Next, L20 shows the highest absorption in the human intestine (1.0000) but has low blood-brain barrier permeability and mixed activity in P-glycoprotein interactions, making it a strong candidate for targeted systemic applications with limited CNS penetration. Ligand L03 demonstrates strong binding affinity against *Phytophthora capsica*, highlighting its potential as an effective fungicide; however, further studies on environmental impact and non-target toxicity are essential for sustainable agricultural use in both AutoDock and Glide simulations. This convergence of results from different molecular docking methodologies emphasizes the reliability and consistency of L03’s binding affinity for this particular fungal pathogen.

## Supporting information

S1 Fig Optimized structure of molecules. (DOCX)

S2 Fig Frontier Molecular Orbital of HOMO-LUMO for optimized structure of molecules. (DOCX)

S3 Fig Electrostatic potential map for optimized structure of molecules. (DOCX)

S4 Fig Molecular docking pose of molecules. (DOCX)

## References

[1] Fones HN, Bebber DP, Chaloner TM, Kay WT, Steinberg G, Gurr SJ. Threats to global food security from emerging fungal and oomycete crop pathogens. Nat Food. 2020;1(6):332–42. doi: 10.1038/s43016-020-0075-0 37128085

[2] Lengai GMW, Muthomi JW, Mbega ER. Phytochemical activity and role of botanical pesticides in pest management for sustainable agricultural crop production. Scientific Afr. 2020;7:e00239. doi: 10.1016/j.sciaf.2019.e00239

[3] Popp J, Pető K, Nagy J. Pesticide productivity and food security. a review. Agron Sustain Dev. 2012;33(1):243–55. doi: 10.1007/s13593-012-0105-x

[4] Inamuddin A, Asiri A, Suvardhan K. Green sustainable process for chemical and environmental engineering and science. Elsevier; 2019.

[5] Bozdogan A. Assessment of total risk on non-target organisms in fungicide application for agricultural sustainability. Sustainability. 2014;6(2):1046–58. doi: 10.3390/su6021046

[6] Zhang Z, Bills GF, An Z. Advances in the treatment of invasive fungal disease. PLoS Pathog. 2023;19(5):e1011322. doi: 10.1371/journal.ppat.1011322 37141208 PMC10159104

[7] Frąc M, Hannula SE, Bełka M, Jędryczka M. Fungal Biodiversity and Their Role in Soil Health. Front Microbiol. 2018;9 707. doi: 10.3389/fmicb.2018.00707 29755421 PMC5932366

[8] Rameez M, Khan N, Ahmad S, Ahmad MM. Bionanocomposites: a new approach for fungal disease management. Biocat Agri Biotech. 2024;57:103115. doi: 10.1016/j.bcab.2024.103115

[9] Hausbeck MK, Lamour KH. Phytophthora capsici on vegetable crops: research progress and management challenges. Plant Dis. 2004;88(12):1292–303. doi: 10.1094/PDIS.2004.88.12.1292 30795189

[10] Romanazzi G, Feliziani E. Botrytis cinerea (gray mold). in Postharvest decay. Elsevier. 2014:131–46.

[11] De Cal A, Pascual S, Larena I, Melgarejo P. Biological control of Fusarium oxysporum f. sp. *lycopersici*. Plant Pathol. 1995;44(5):909–17. doi: 10.1111/j.1365-3059.1995.tb02750.x

[12] Roelfs AP. An international system of nomenclature for Puccinia graminisf. sp.*tritici*. Phytopathol. 1988;78(5):526. doi: 10.1094/phyto-78-526

[13] Saberi Riseh R, Vatankhah M, Hassanisaadi M, Barka EA. Unveiling the role of hydrolytic enzymes from soil biocontrol bacteria in sustainable phytopathogen management. Front Biosci (Landmark Ed). 2024;29(3):105. doi: 10.31083/j.fbl2903105 38538262

[14] Adisa IO. Potential of nanoscale elements to control Fusarium wilt disease in tomato (Solanum lycopersicum), enhance macronutrient use efficiency, and increase its yield. The University of Texas at El Paso; 2019.

[15] Garvey M, Rowan NJ. Pathogenic drug resistant fungi: a review of mitigation strategies. Int J Mol Sci. 2023;24(2):1584. doi: 10.3390/ijms24021584 36675092 PMC9863602

[16] Kawsar SMA, Almalki FA, Hadd TB, Laaroussi H, Khan MAR, Hosen MA, et al. Potential antifungal activity of novel carbohydrate derivatives validated by POM, molecular docking and molecular dynamic simulations analyses. Molecular Simula. 2022;49(1):60–75. doi: 10.1080/08927022.2022.2123948

[17] Rathod S, Bhande D, Pawar S, Gumphalwad K, Choudhari P, More H. Identification of potential hits against fungal lysine deacetylase Rpd3 via molecular docking, molecular dynamics simulation, DFT, In-Silico ADMET and drug-likeness assessment. Chem Afr. 2023;7(2):1151–64. doi: 10.1007/s42250-023-00766-5

[18] Singh B, et al. Evaluating terrestrol a as an inhibitor against SARS‐CoV‐2 and invasive fungal pathogens: a comprehensive computational analysis. Chem Select. 2024;9(14):e202304761.

[19] Xu J. Assessing global fungal threats to humans. mLife. 2022;1(3):223–40. doi: 10.1002/mlf2.12036 38818220 PMC10989982

[20] Sudhakar C, Sawant K, Kumar S, Shukla M, Rana M, Srivastava S. Brown spot: an embryonic disease of rice; 2022.

[21] Ahn I-P. Glufosinate ammonium-induced pathogen inhibition and defense responses culminate in disease protection in bar-transgenic rice. Plant Physiol. 2008;146(1):213–27. doi: 10.1104/pp.107.105890 17981989 PMC2230565

[22] Vurro M, Bonciani B, Vannacci G. Emerging infectious diseases of crop plants in developing countries: impact on agriculture and socio-economic consequences. Food Sec. 2010;2(2):113–32. doi: 10.1007/s12571-010-0062-7

[23] Lal M, Sharma S, Yadav S, Kumar S. Management of late blight of potato. Potato-from Incas to All Over the World. 2018:83–106.

[24] Umadevi P, Anandaraj M. Proteomic analysis of the tripartite interaction between black pepper, Trichoderma harzianum and Phytophthora capsici provides insights into induced systemic resistance mediated by Trichoderma spp.. Eur J Plant Pathol. 2019;154(3):607–20. doi: 10.1007/s10658-019-01685-3

[25] Hua GKH, Ji P, Culbreath AK, Ali ME. Comparative study of phosphorous-acid-containing products for managing phytophthora blight of bell pepper. Agronomy. 2022;12(6):1293. doi: 10.3390/agronomy12061293

[26] Hannukkala AO. Examples of alien pathogens in Finnish potato production-their introduction, establishment and consequences; 2011.

[27] Cayanan DF. Sensitivity of nursery plants to free chlorine in the management of plant pathogens in irrigation water. University of Guelph; 2008.

[28] Lozada DN, Nunez G, Lujan P, Dura S, Coon D, Barchenger DW, et al. Genomic regions and candidate genes linked with Phytophthora capsici root rot resistance in chile pepper (Capsicum annuum L.). BMC Plant Biol. 2021;21(1):601. doi: 10.1186/s12870-021-03387-7 34922461 PMC8684135

[29] Govrin EM, Levine A. The hypersensitive response facilitates plant infection by the necrotrophic pathogen Botrytis cinerea. Curr Biol. 2000;10(13):751–7. doi: 10.1016/s0960-9822(00)00560-1 10898976

[30] Chen X, Wang Y, Gao Y, Gao T, Zhang D. Inhibitory abilities of bacillus isolates and their culture filtrates against the gray mold caused by botrytis cinerea on postharvest fruit. Plant Pathol J. 2019;35(5):425–36. doi: 10.5423/PPJ.OA.03.2019.0064 31632218 PMC6788410

[31] Li T, Liu R, Liu Z, Chang J, Li J. Effects of intermittent temperature and humidity regulation on tomato gray mold. Plant Dis. 2023;107(8):2335–45. doi: 10.1094/PDIS-10-22-2339-RE 36627805

[32] Leyronas C, Fatnassi H, Bardin M, Boulard T, Nicot PC. Modelling botrytis cinerea spore exchanges and production in unheated greenhouses. J Plant Pathol. 2011:407–14.

[33] Kulkarni SJ. An insight into research and investigations of gray mold focused on Botrytis cinerea. in Driving factors for venture creation and success in agricultural entrepreneurship. 2022:273–89.

[34] Sammonds J. Pre-infection processes of Botryosphaeriaceae species conidia. Lincoln University; 2015.

[35] Elmer PA, Michailides TJ. Epidemiology of Botrytis cinerea in orchard and vine crops. in Botrytis: Biology, Pathology and Control. Springer 2007:243–72.

[36] Poveda J, Barquero M, González-Andrés F. Insight into the microbiological control strategies against botrytis cinerea using systemic plant resistance activation. Agronomy. 2020;10(11):1822. doi: 10.3390/agronomy10111822

[37] Davidson JA, Pande S, Bretag TW, Lindbeck KD, Krishna-Kishore G. Biology and management of Botrytis spp. in legume crops. in Botrytis: biology, pathology and control. 2007:295–318.

[38] Raynaldo FA, Xu Y, , Wang Q, Wu B, Li D. Biological control and other alternatives to chemical fungicides in controlling postharvest disease of fruits caused by <i>Alternaria alternata</i> and <i>Botrytis cinerea</i>. Food Innov Adv. 2024;3(2):135–43. doi: 10.48130/fia-0024-0014

[39] Bojkov G. Gray Mold (Botrytis Cinerea) at Vines. USA: Scientific Research Publishing, Inc; 2022.

[40] Wakeham A, Langton A, Adams S, Kennedy R. Interface of the environment and occurrence of Botrytis cinerea in pre-symptomatic tomato crops. Crop Protect. 2016;90:27–33. doi: 10.1016/j.cropro.2016.08.014

[41] Maurya S, Dubey S, Kumari R, Verma R. Management tactics for fusarium wilt of tomato caused by Fusarium oxysporum f. sp. *lycopersici* (Sacc.): a review. Management. 2019;4(5):1–7.

[42] Kavroulakis N, Ntougias S, Besi MI, Katsou P, Damaskinou A, Ehaliotis C, et al. Antagonistic bacteria of composted agro-industrial residues exhibit antibiosis against soil-borne fungal plant pathogens and protection of tomato plants from Fusarium oxysporum f.sp. radicis-lycopersici. Plant Soil. 2010;333(1–2):233–47. doi: 10.1007/s11104-010-0338-x

[43] Kumari SMP, Prabina BJ. Protection of tomato, Lycopersicon esculentum from wilt pathogen, Fusarium oxysporum f. sp. *lycopersici* by arbuscular mycorrhizal fungi, Glomus sp. Int J Curr Microbiol Appl Sci. 2019;8:1368–78.

[44] Bawa I. Management strategies of Fusarium wilt disease of tomato incited by Fusarium oxysporum f. sp. *lycopersici* (Sacc.) a review. Int J Adv Acad Res. 2016;2(5).

[45] Srinivas C, Nirmala Devi D, Narasimha Murthy K, Mohan CD, Lakshmeesha TR, Singh B, et al. Fusarium oxysporum f. sp. *lycopersici* causal agent of vascular wilt disease of tomato: Biology to diversity- a review. Saudi J Biol Sci. 2019;26(7):1315–24. doi: 10.1016/j.sjbs.2019.06.002 31762590 PMC6864208

[46] Devi S. 14. Fusarium wilt of brinjal. n.d.

[47] Thatcher LF, Kidd BN, Kazan K. Belowground defence strategies against Fusarium oxysporum. Belowground defence strategies in plants. 2016:71–98.

[48] López-Zapata S, García-Jaramillo DJ, López WR, Ceballos-Aguirre N. Tomato (Solanum lycopersicum L.) and Fusarium oxysporum f. sp. *lycopersici* interaction. a review. Revista UDCA Actualidad & Divulgación Científica. 2021;24(1).

[49] Beyan A, Seid A, Shifa H. Response of tomato genotypes to Meloidogyne javanica and Fusarium oxysporum f. sp. *lycopersici* co-infestation under glasshouse conditions; 2019.

[50] Gao F, Wu X, Sun H, Wang Z, Chen S, Zou L, et al. Identification of Stem Rust Resistance Genes in Triticum Wheat Cultivars and Evaluation of Their Resistance to Puccinia graminis f. sp. *tritici*. Agriculture. 2024;14(2):198. doi: 10.3390/agriculture14020198

[51] Brueggeman RS, Solanki S, Ameen G, Effertz K, Poudel RS, Karakaya A. Fungal diseases affecting barley. in Achieving sustainable cultivation of barley. Burleigh Dodds Science Publishing. 2020:265–322.

[52] Alkhalifah DHM, Damra E, Melhem MB, Hozzein WN. Fungus under a changing climate: modeling the current and future global distribution of fusarium oxysporum using geographical information system data. Microorganisms. 2023;11(2):468. doi: 10.3390/microorganisms11020468 36838433 PMC9967672

[53] Ontoy JC, Ham JH. Mapping and omics integration: towards precise rice disease resistance breeding. Plants. 2024;13(9):1205. 38732420 10.3390/plants13091205 PMC11085595

[54] Kohn W, Becke AD, Parr RG. Density functional theory of electronic structure. J Phys Chem. 1996;100(31):12974–80. doi: 10.1021/jp960669l

[55] Fukui K, Pullman B. Horizons of quantum chemistry. Springer; 1980. doi: 10.1007/978-94-009-9027-2

[56] Parr RG, Yang W. Density functional approach to the frontier-electron theory of chemical reactivity. J Am Chem Soc. 1984;106(14):4049–50. doi: 10.1021/ja00326a036

[57] Yang C, Huang Z, Zhang X, Zhu C. Structural Insights into the NAD(P)H:Quinone Oxidoreductase from Phytophthora capsici. ACS Omega. 2022;7(29):25705–14. doi: 10.1021/acsomega.2c02954 35910145 PMC9330140

[58] Lou T, et al. Structural insights into three sesquiterpene synthases for the biosynthesis of tricyclic sesquiterpenes and chemical space expansion by structure-based mutagenesis. J Am Chem Soc. 2023;145(15):8474–85. 10.1021/jacs.3c00278 37018048

[59] Yu DS. The structural repertoire of Fusarium oxysporum f. sp. *lycopersici* effectors revealed by experimental and computational studies. BioRxiv. 2021. 10.7554/eLife.89280 PMC10942635 38411527

[60] Zhao Y-B, Liu M-X, Chen T-T, Ma X, Li Z-K, Zheng Z, et al. Pathogen effector AvrSr35 triggers Sr35 resistosome assembly via a direct recognition mechanism. Sci Adv. 2022;8(36):eabq5108. doi: 10.1126/sciadv.abq5108 36083908 PMC9462685

[61] Spassov V, Yan L. Accelrys software inc., discovery studio modeling environment, release 4.0. Proteins: Str, Fun, and Bioinform. 2013;81:704–14.

[62] Sándor M, Kiss R, Keseru GM. Virtual fragment docking by Glide: a validation study on 190 protein-fragment complexes. J Chem Inf Model. 2010;50(6):1165–72. doi: 10.1021/ci1000407 20459088

[63] Tsaioun K, Blaauboer BJ, Hartung T. Evidence-based absorption, distribution, metabolism, excretion (ADME) and its interplay with alternative toxicity methods. ALTEX. 2016;33(4):343–58. doi: 10.14573/altex.1610101 27806179

[64] Chandrasekaran SB, Abed N, Al-Attraqchi O, Kuche K, Tekade RK. Computer-aided prediction of pharmacokinetic (ADMET) properties. in Dosage Form Design Parameters. 2018:731–55.

[65] Pires DEV, Blundell TL, Ascher DB. pkCSM: predicting small-molecule pharmacokinetic and toxicity properties using graph-based signatures. J Med Chem. 2015;58(9):4066–72. doi: 10.1021/acs.jmedchem.5b00104 25860834 PMC4434528

[66] Daina A, Michielin O, Zoete V. SwissADME: a free web tool to evaluate pharmacokinetics, drug-likeness and medicinal chemistry friendliness of small molecules. Sci Rep. 2017;7:42717. doi: 10.1038/srep42717 28256516 PMC5335600

[67] Bowers KJ. Scalable algorithms for molecular dynamics simulations on commodity clusters. in Proceedings of the 2006 ACM/IEEE Conference on Supercomputing. 2006:84-es.

[68] López J, Anitescu C, Valizadeh N, Rabczuk T, Alajlan N. Structural shape optimization using Bézier triangles and a CAD-compatible boundary representation. Eng Comp. 2019;36(4):1657–72. doi: 10.1007/s00366-019-00788-z

[69] Kumer A, Ahmed B, Sharif M, Al-Mamun A. A theoretical study of aniline and nitrobenzene by computational overview. Asian J Phy Chem Sci. 2017;4(2):1–12.

[70] Kumer A, Paul S, Sarker MN, Islam MJ. The prediction of thermo physical, vibrational spectroscopy, chemical reactivity, biological properties of morpholinium borate, phosphate, chloride and bromide ionic liquid: a DFT study. Inter J New Chem. 2019;6(4):236–53.

[71] Kumer A, Sarker MN, Paul S. The theoretical investigation of HOMO, LUMO, thermophysical properties and QSAR study of some aromatic carboxylic acids using HyperChem programming. Intern J Chem Tech. 2019;3(1):26–37. doi: 10.32571/ijct.478179

[72] Kumer A, Sarker MN, Paul S, Zannat A. The theoretical prediction of thermophysical properties, HOMO, LUMO, QSAR and Biological Indics of Cannabinoids (CBD) and Tetrahhdrocannabinol (THC) by computational chemistry. Adv J Chem A. 2019;2(3):190–202. doi: 10.33945/sami/ajca.2019.2.190202

[73] Akash S, Aovi FI, Azad MAK, Kumer A, Chakma U, Islam MR, et al. A drug design strategy based on molecular docking and molecular dynamics simulations applied to development of inhibitor against triple-negative breast cancer by Scutellarein derivatives. PLoS One. 2023;18(10):e0283271. doi: 10.1371/journal.pone.0283271 37824496 PMC10569544

[74] Hashem HE, Ahmad S, Kumer A, Bakri YE. In silico and in vitro prediction of new synthesized N-heterocyclic compounds as anti-SARS-CoV-2. Sci Rep. 2024;14(1):1152. doi: 10.1038/s41598-024-51443-7 38212472 PMC10784557

[75] Kobir ME, Ahmed A, Roni MAH, Chakma U, Amin MR, Chandro A, et al. Anti-lung cancer drug discovery approaches by polysaccharides: an in silico study, quantum calculation and molecular dynamics study. J Biomol Struct Dyn. 2023;41(14):6616–32. doi: 10.1080/07391102.2022.2110156 35968631

[76] Rani M, Nath A, Kumer A. In-silico investigations on the anticancer activity of selected 2-aryloxazoline derivatives against breast cancer. J Biomol Struct Dyn. 2023;41(17):8392–401. doi: 10.1080/07391102.2022.2134208 36245134

[77] Kawsar S, Kumer A. Computational investigation of methyl α-D-glucopyranoside derivatives as inhibitor against bacteria, fungi and COVID-19 (SARS-2). J Chile Chem Soc. 2021;66(2):5206–14.

[78] Kumer A, Khan MW. The effect of alkyl chain and electronegative atoms in anion on biological activity of anilinium carboxylate bioactive ionic liquids and computational approaches by DFT functional and molecular docking. Heliyon. 2021;7(7):e07509. doi: 10.1016/j.heliyon.2021.e07509 34296013 PMC8282962

[79] Kumer A, Khan MW. Synthesis, characterization, antimicrobial activity and computational exploration of ortho toludinium carboxylate ionic liquids. J Molecular Str. 2021;1245:131087. doi: 10.1016/j.molstruc.2021.131087

[80] Hoque M, Kumer A, Hussen MS, Khan MW. Theoretical evaluation of 5,6-diaroylisoindoline-1,3-dione as potential carcinogenic kinase PAK1 inhibitor: DFT calculation, molecular docking study and ADMET prediction. Inte J Adv Biologi Biomedi Res. 2021;9(1):77–104.

[81] Islam MJ, Sarker MN, Kumer A, Paul S. The evaluation and comparison of thermo-physical, chemical and biological properties of palladium (II) complexes on binuclear amine ligands with different anions by DFT study. Inte J Adv Biologi Biomedi Res. 2019;7(4):315–34.

[82] Kumer A, Chakma U, Chandro A, Howlader D, Akash S, Kobir MdE, et al. Modified D-Glucofuranose computationally screening for inhibitor of breast cancer and triple breast cancer: chemical descriptor, molecular docking, molecular dynamics And QSAR. J Chil Chem Soc. 2022;67(3):5623–35. doi: 10.4067/s0717-97072022000305623

[83] Kumer A, Chakma U, Rana MM, Chandro A, Akash S, Elseehy MM, et al. Investigation of the new inhibitors by sulfadiazine and modified derivatives of α-D-glucopyranoside for white spot syndrome virus disease of shrimp by. In Silico: Quantum Calculations, Molecular Docking, ADMET and Molecular Dynamics Study. Molecules. 2022;27(12):3694. doi: 10.3390/molecules27123694 35744817 PMC9228161

[84] Kumer A, Jahidul M, Paul S. Effect of external electric field and temperature on entropy, heat of capacity, and chemical reactivity with QSAR study of morphonium chloride and nitrous ionic liquids crystal using DFT. Chem Methodolo. 2020;4:595–604.

[85] Cheng K, Zheng Q-Z, Qian Y, Shi L, Zhao J, Zhu H-L. Synthesis, antibacterial activities and molecular docking studies of peptide and Schiff bases as targeted antibiotics. Bioorg Med Chem. 2009;17(23):7861–71. doi: 10.1016/j.bmc.2009.10.037 19884012

[86] Hermann T, Westhof E. Docking of cationic antibiotics to negatively charged pockets in RNA folds. J Med Chem. 1999;42(7):1250–61. doi: 10.1021/jm981108g 10197968

[87] Athar M, Sona AN, Bekono BD, Ntie-Kang F. Fundamental physical and chemical concepts behind “drug-likeness” and “natural product-likeness”. Phys Sci Rev. 2019;4(12):. doi: 10.1515/psr-2018-0101

[88] Lipinski CA, Lombardo F, Dominy BW, Feeney PJ. Experimental and computational approaches to estimate solubility and permeability in drug discovery and development settings. Adv Drug Deli Rev. 1997;23(1–3):3–25. 10.1016/s0169-409x(00)00129-0 11259830

