# Supplementary figures and images for: Computational and In silico study of novel fungicides against combating root rot, gray mold, fusarium wilt, and cereal rust
Source: PLoS One. 2025 Jan 31;20(1):e0316606. doi: 10.1371/journal.pone.0316606 (PMC11785347; doi:10.1371/journal.pone.0316606)

**S1 Fig.** Optimized structure of molecules.

| **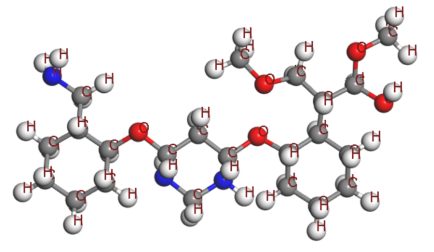** | **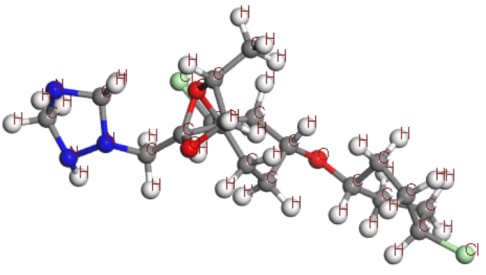** | | **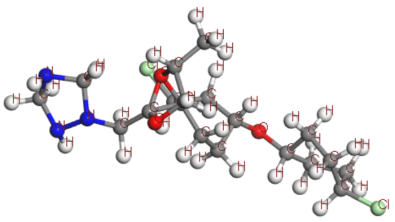** |
| --- | --- | --- | --- |
| **L01** | **L02** | | **L03** |
| **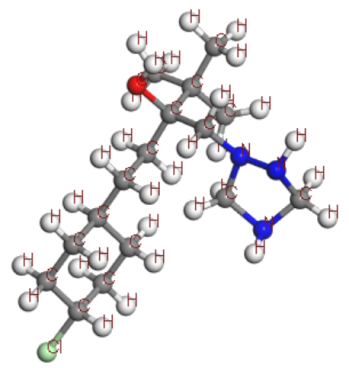** | **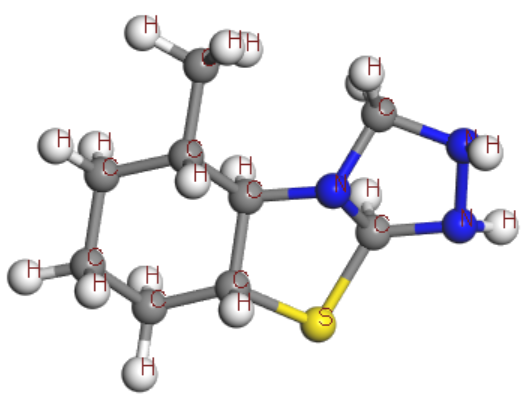** | | **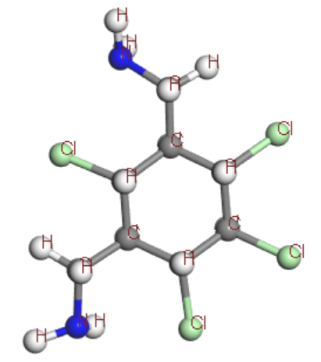** |
| **L04** | **L05** | | **L06** |
| **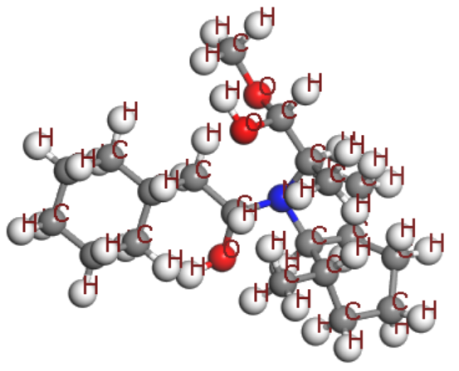** | **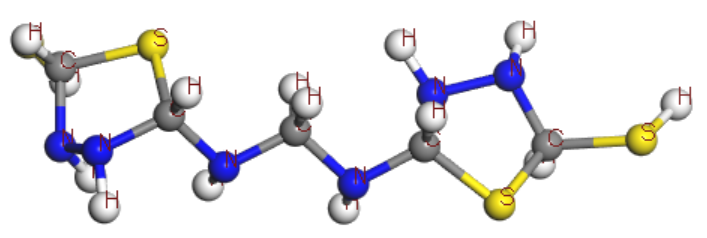** | | **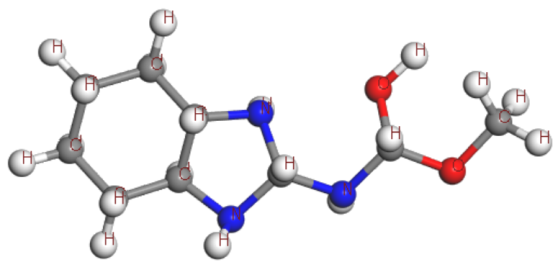** |
| **L07** | **L08** | | **L09** |
| **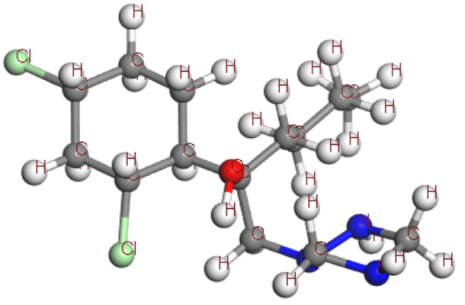** | **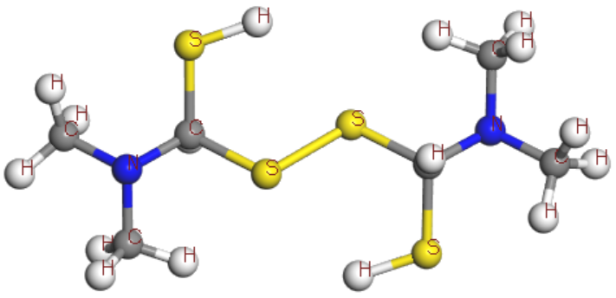** | | **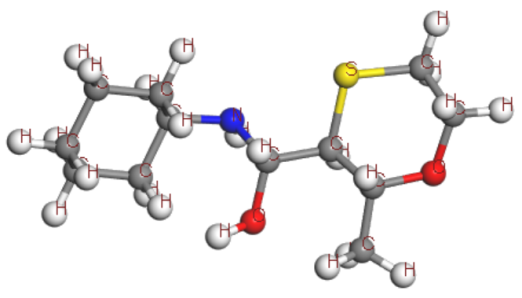** |
| **L010** | **L011** | | **L012** |
| **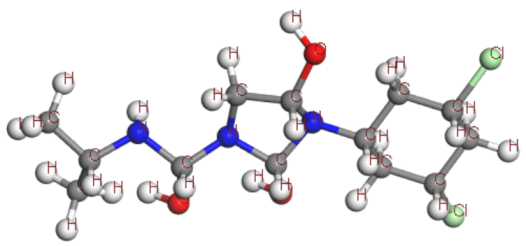** | **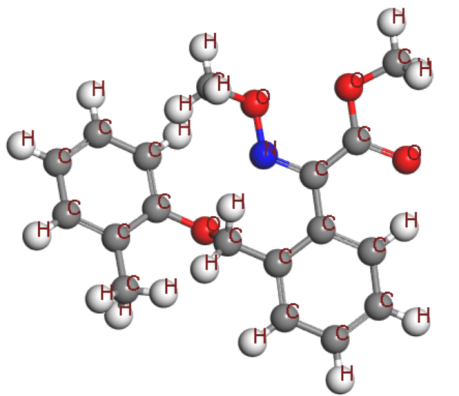** | | **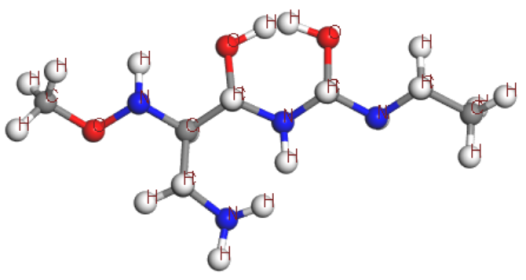** |
| **L013** | **L014** | | **L015** |
| **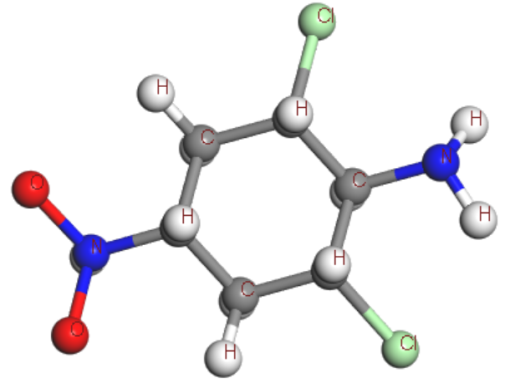** | **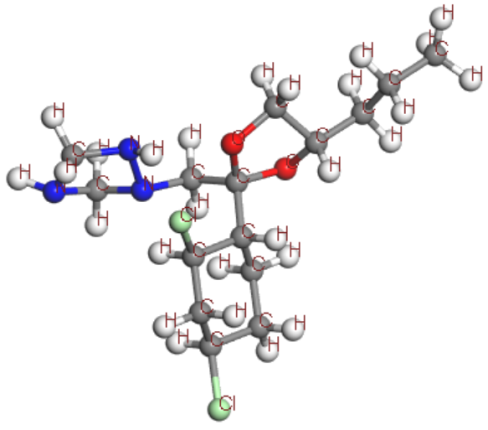** | | **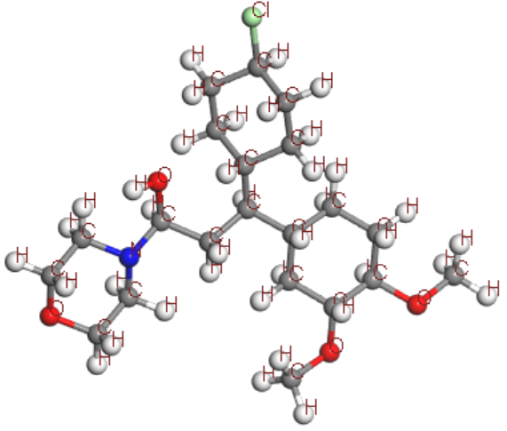** |
| **L016** | **L017** | | **L018** |
| **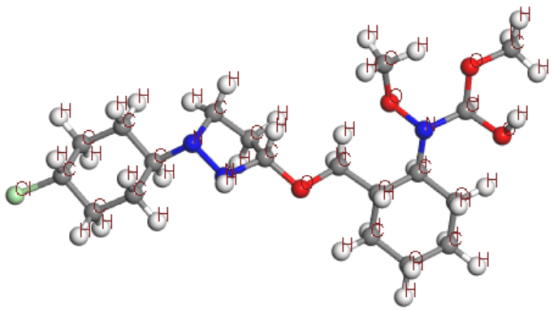** | | **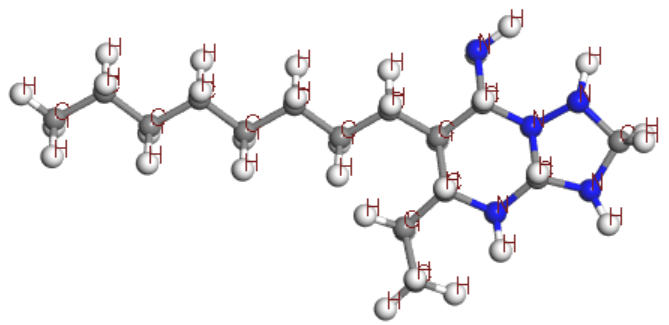** | |
| **L019** | | **L020** | |

Supplement: S1 Fig — (DOCX) [file pone.0316606.s001.docx]
