# Supplementary material for: Computational and In silico study of novel fungicides against combating root rot, gray mold, fusarium wilt, and cereal rust
Source: PLoS One. 2025 Jan 31;20(1):e0316606. doi: 10.1371/journal.pone.0316606 (PMC11785347; doi:10.1371/journal.pone.0316606)
Supplement: S2 Fig — (DOCX) [file pone.0316606.s002.docx]

**S2 Fig**. Frontier Molecular Orbital of HOMO-LUMO for Optimized structure of molecules.

|  | **L01** | **L02** | **L03** |
| --- | --- | --- | --- |
| **LUMO** | **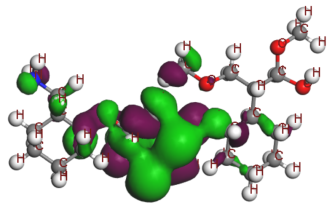** | **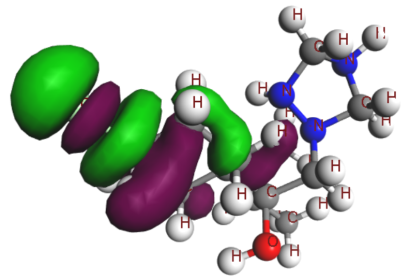** | **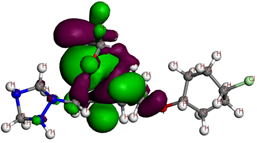** |
| **HOMO** | **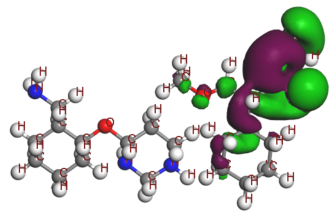** | **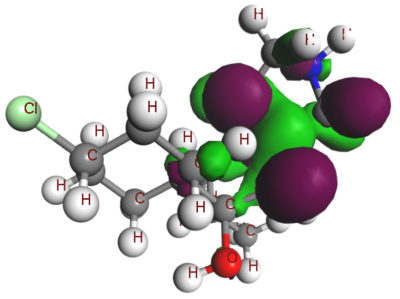** | **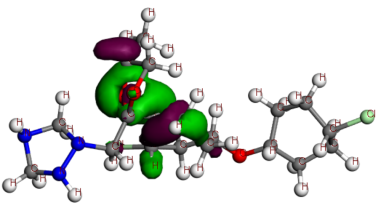** |
|  | **L04** | **L05** | **L06** |
| **LUMO** | **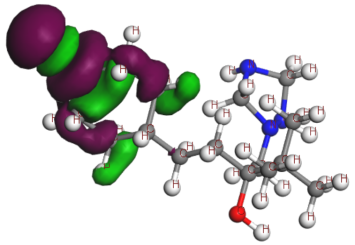** | **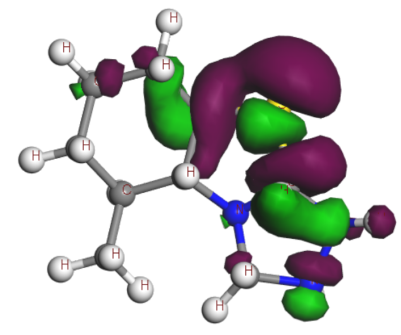** | **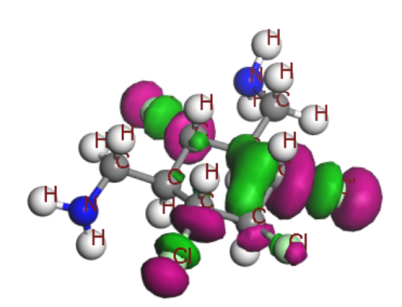** |
| **HOMO** | **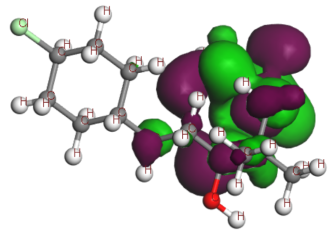** | **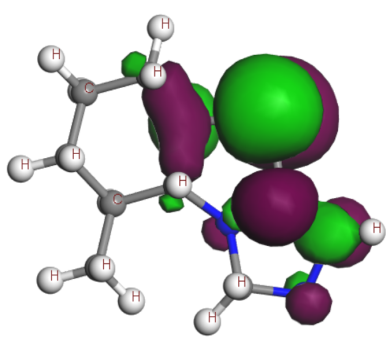** | **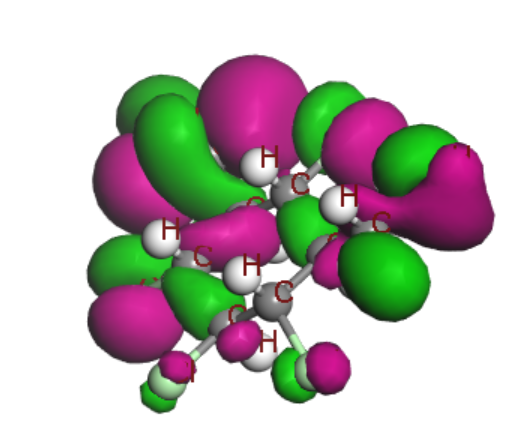** |
|  | **L07** | **L08** | **L09** |
| **LUMO** | **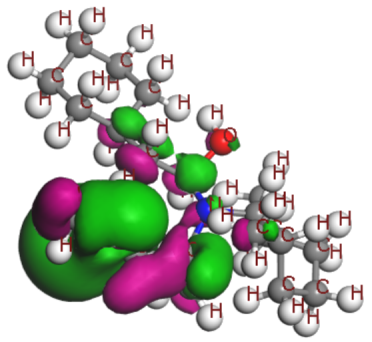** | **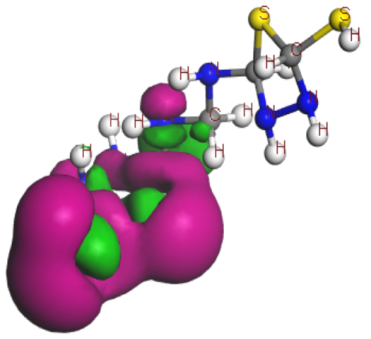** | **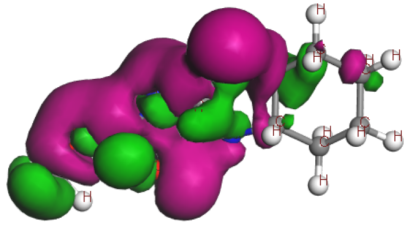** |
| **HOMO** | **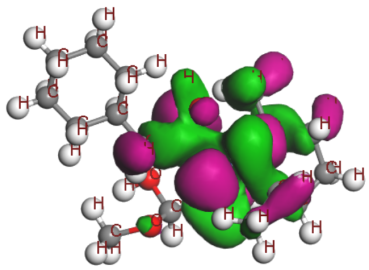** | **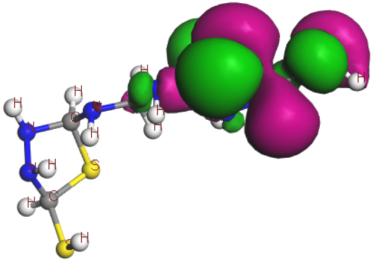** | **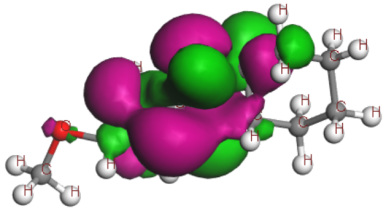** |
|  | **L10** | **L11** | **L12** |
| **LUMO** | **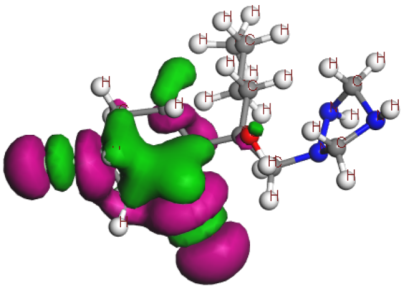** | **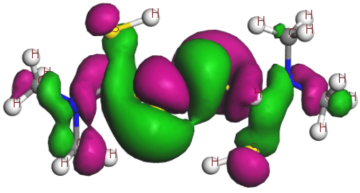** | **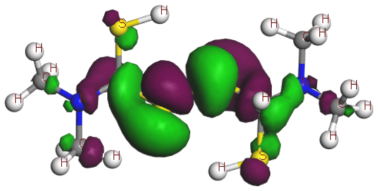** |
| **HOMO** | **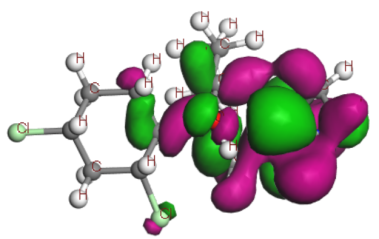** | **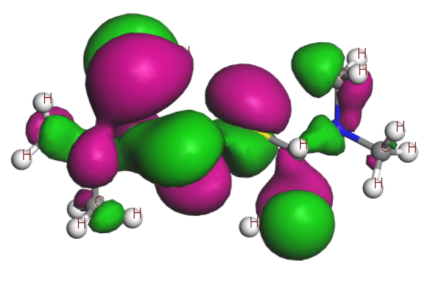** | **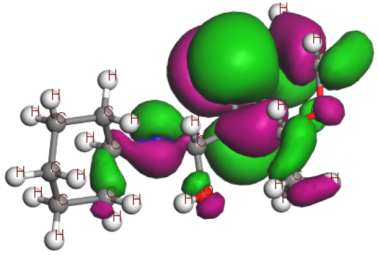** |
|  | **L13** | **L14** | **L15** |
| **LUMO** | **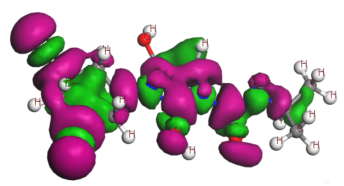** | **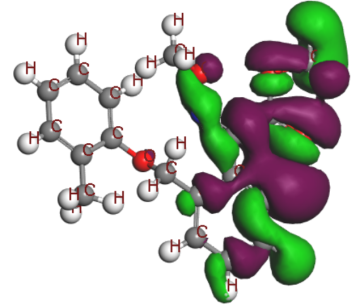** | **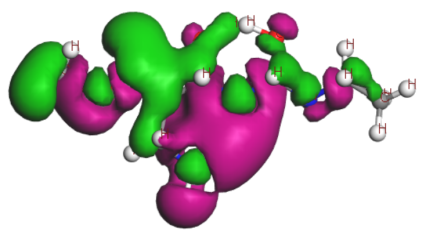** |
| **HOMO** | **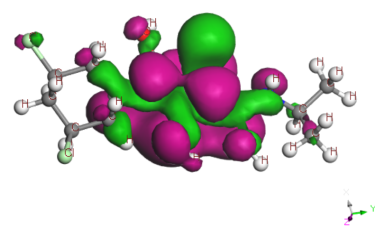** | **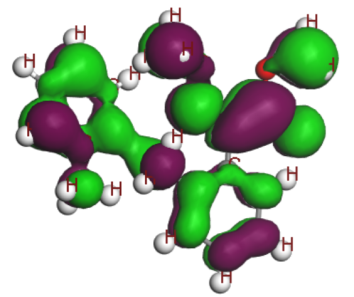** | **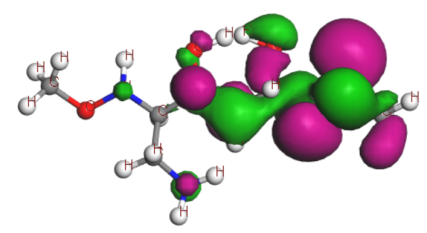** |
|  | **L16** | **L17** | **L18** |
| **LUMO** | **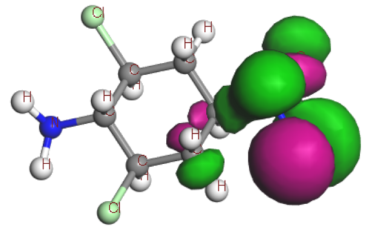** | **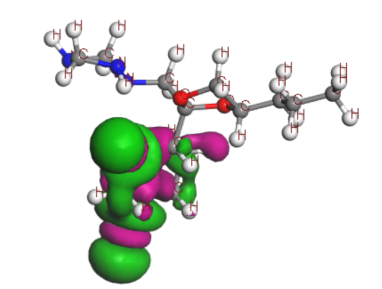** | **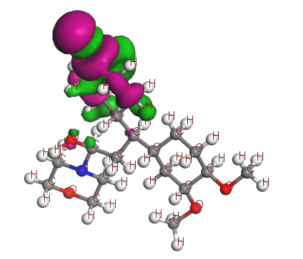** |
| **HOMO** | **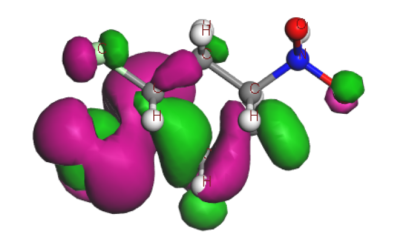** | **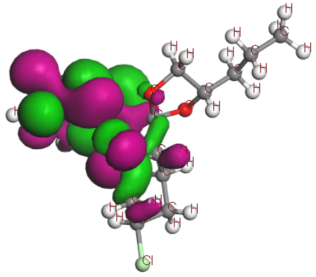** | **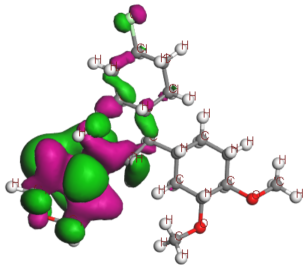** |
|  | **L19** | **L20** |  |
| **LUMO** | **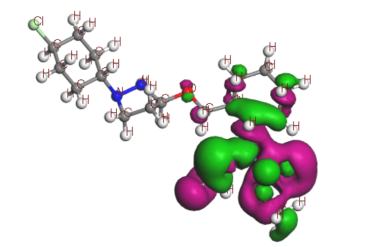** | **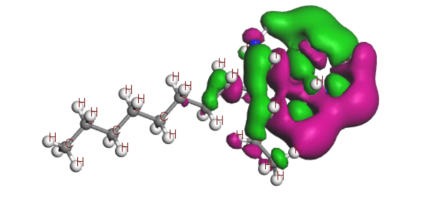** |  |
| **HOMO** | **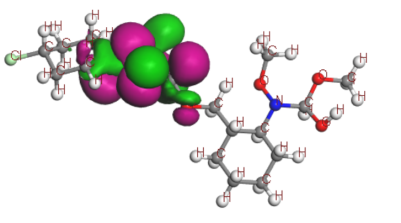** | **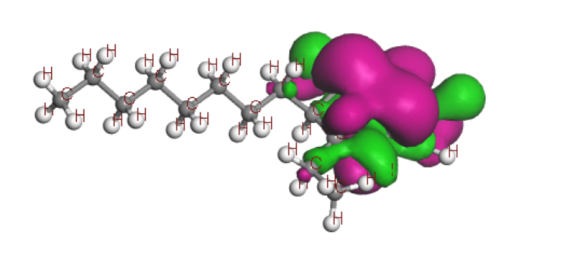** |  |
