# Supplementary material for: Computational and In silico study of novel fungicides against combating root rot, gray mold, fusarium wilt, and cereal rust
Source: PLoS One. 2025 Jan 31;20(1):e0316606. doi: 10.1371/journal.pone.0316606 (PMC11785347; doi:10.1371/journal.pone.0316606)
Supplement: S3 Fig — (DOCX) [file pone.0316606.s003.docx]

**S3 Fig**. Electrostatic Potential Map for Optimized structure of molecules.

| **L01** | **L02** |
| --- | --- |
| **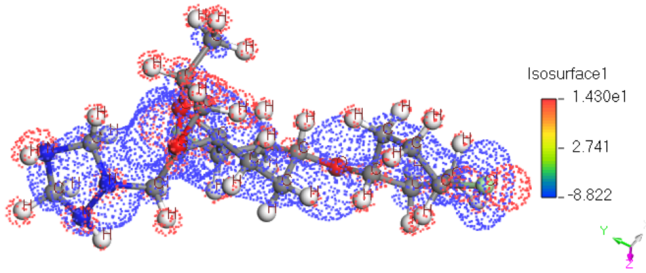** | **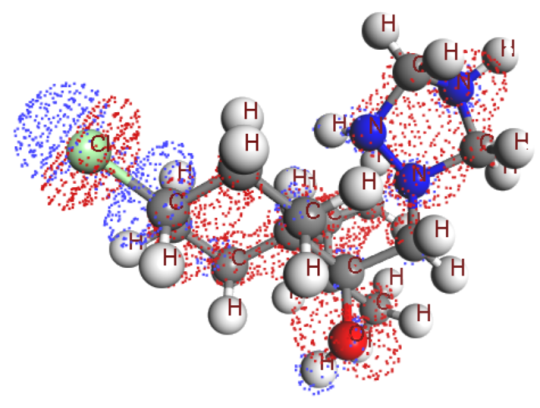** |
| **L03** | **L04** |
| **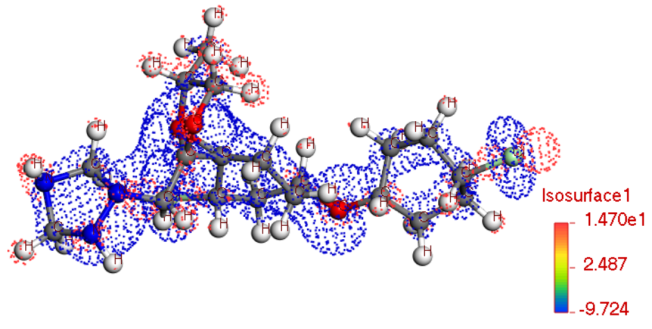** | **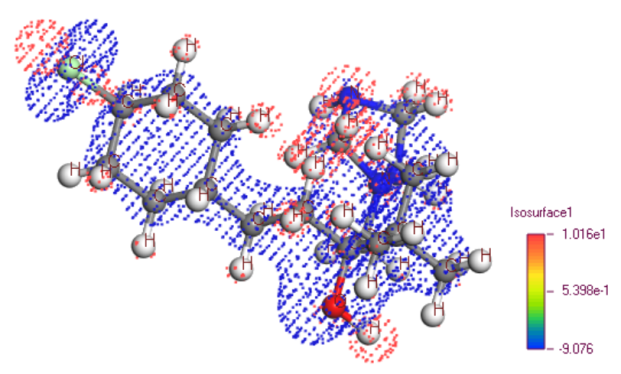** |
| **L05** | **L06** |
| **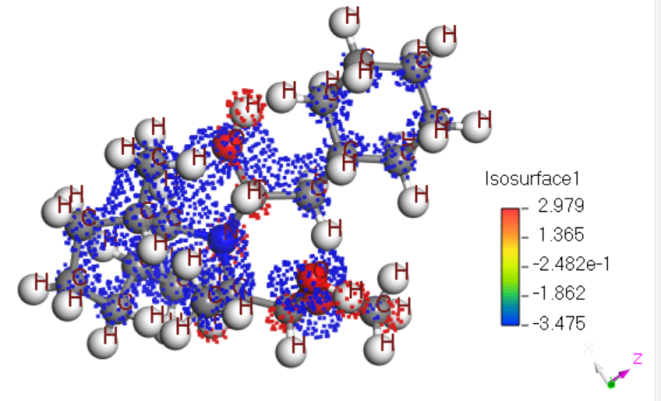** | **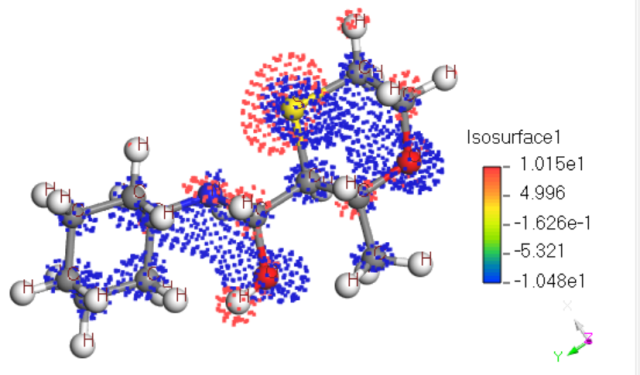** |
| **L07** | **L08** |
| **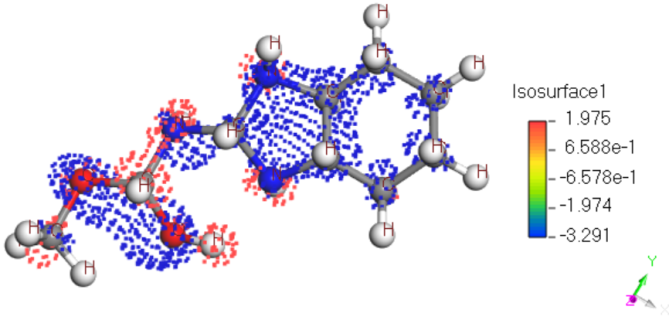** | **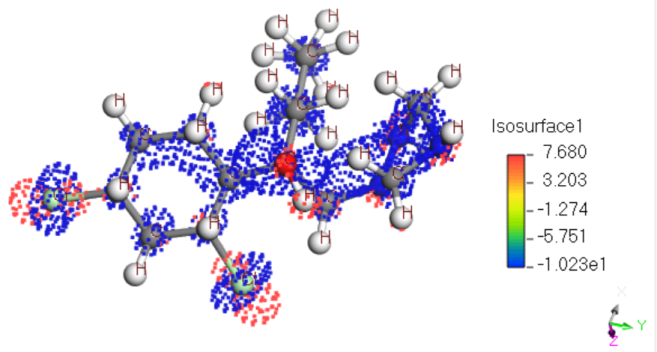** |
| **L09** | **L10** |
| **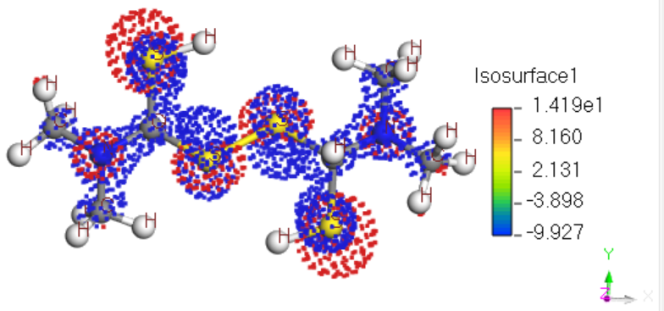** | **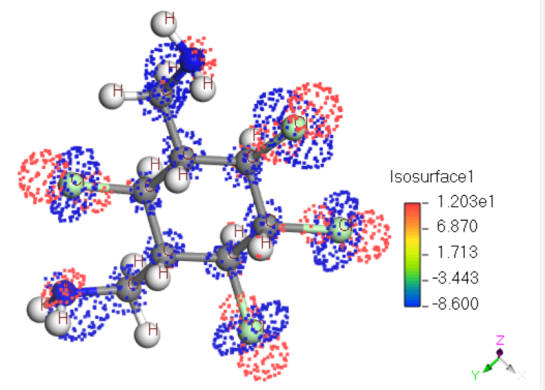** |
| **L11** | **L12** |
| **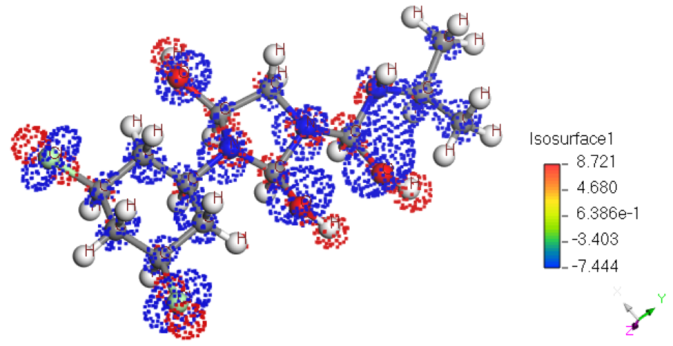** | **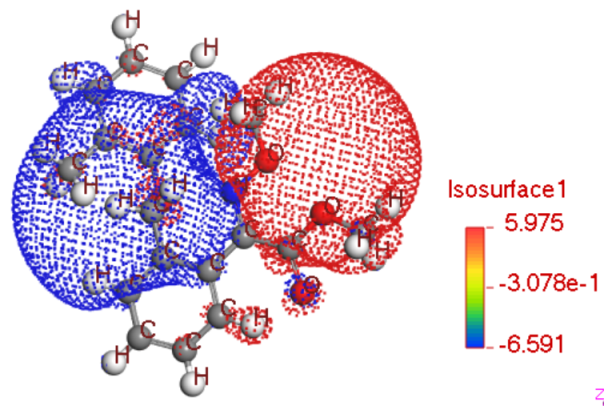** |
| **L13** | **L14** |
| **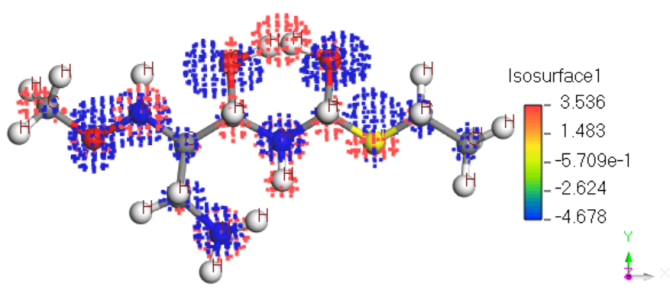** | **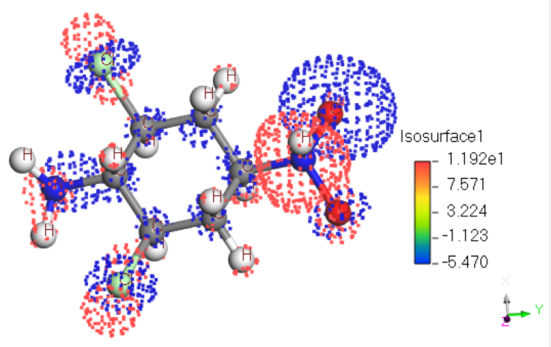** |
| **L15** | **L16** |
| **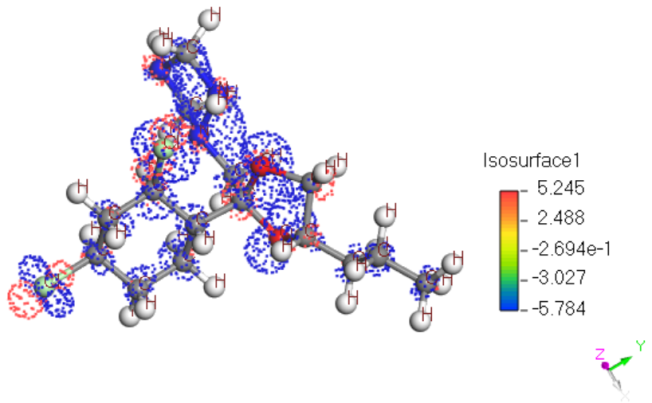** | **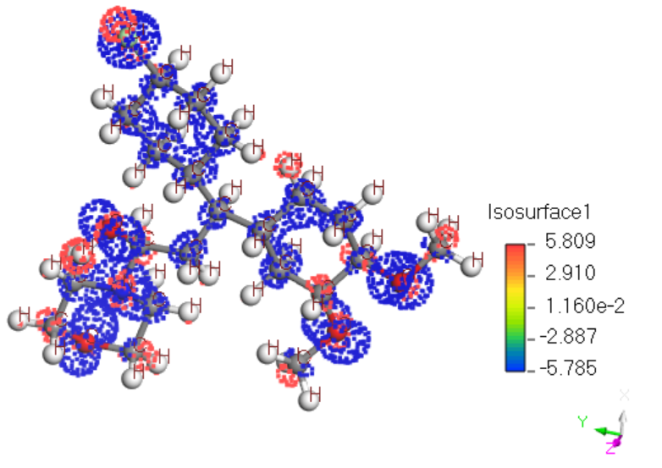** |
| **L17** | **L18** |
| **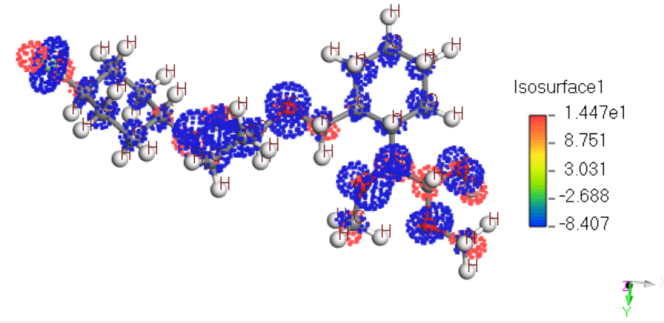** | **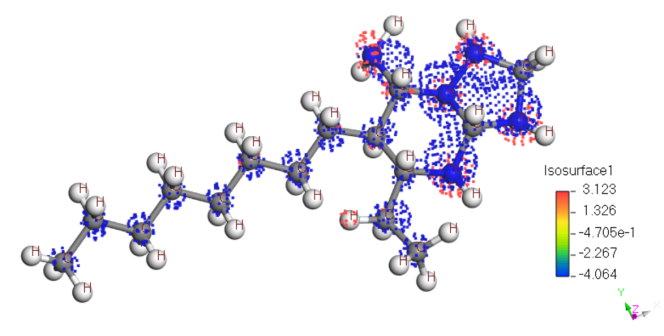** |
| **L19** | **L20** |
