# Supplementary material for: Computational and In silico study of novel fungicides against combating root rot, gray mold, fusarium wilt, and cereal rust
Source: PLoS One. 2025 Jan 31;20(1):e0316606. doi: 10.1371/journal.pone.0316606 (PMC11785347; doi:10.1371/journal.pone.0316606)
Supplement: S4 Fig — (DOCX) [file pone.0316606.s004.docx]

**S4 Fig.** Molecular docking pose of molecules.

| *1_L03_pythopthora1_*  *PyRx_AutoDock4* | 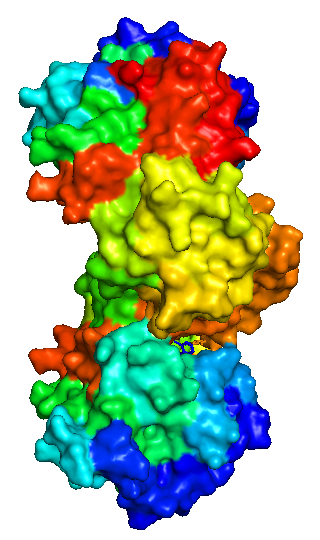 | 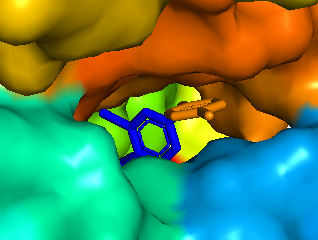 | 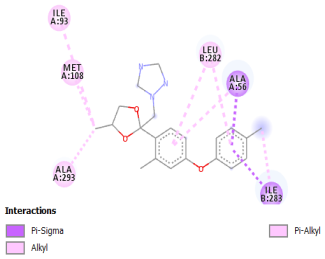 | 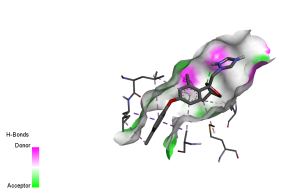 |
| --- | --- | --- | --- | --- |
| 2_L03_sclerotium2_  PyRx_AutoDock4 | 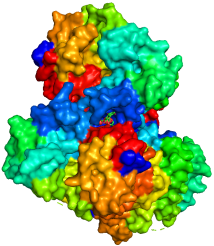 | 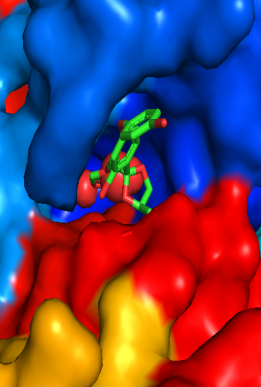 | 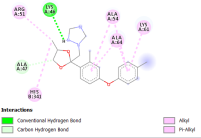 | 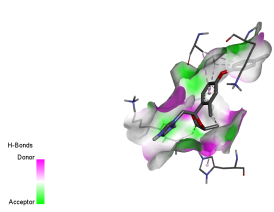 |
| 3_L03_fusarium3_  PyRx_AutoDock4 | 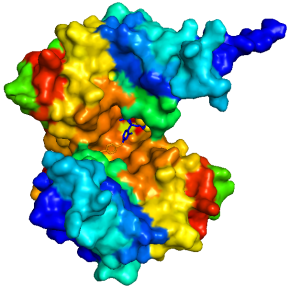 | 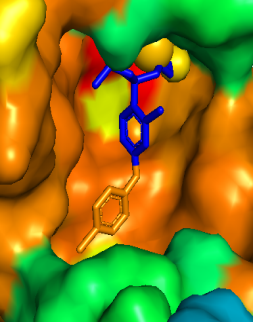 | 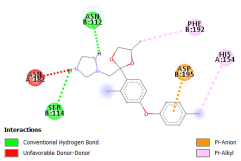 | 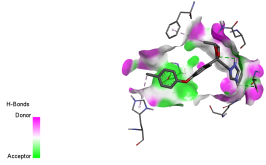 |
| 4_L03_pucinia4_  PyRx_AutoDock4 | 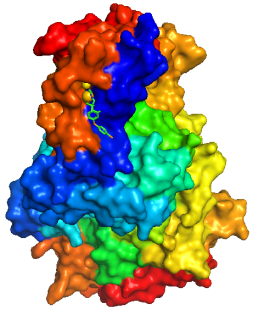 | 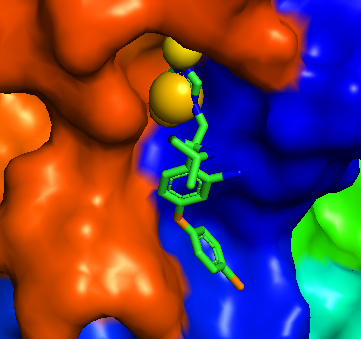 | 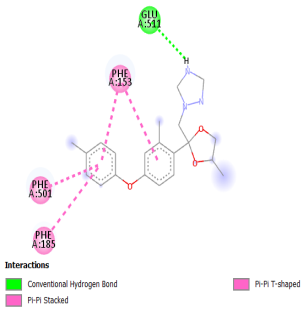 | 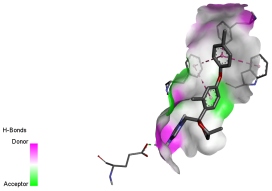 |
| 5_L019_Fusarium3_  PyRx_AutoDock4 | 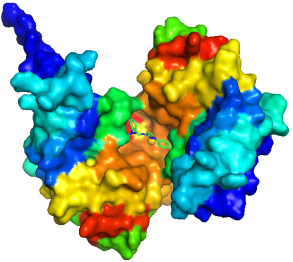 | 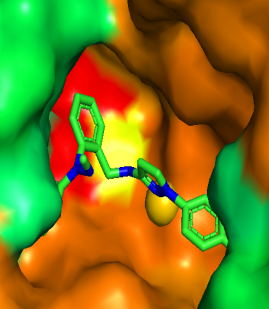 | 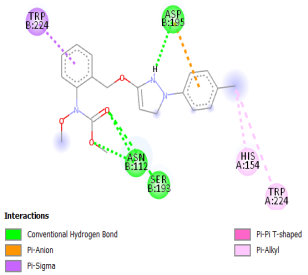 | 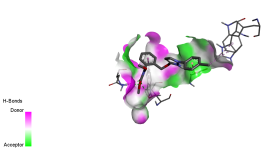 |
| 5_L019_puccinia4_  PyRx_AutoDock4 error | 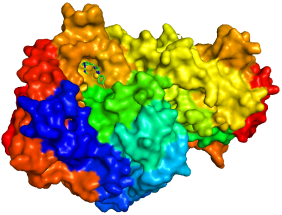 | 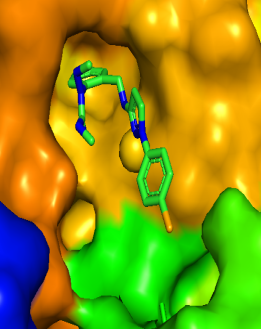 | 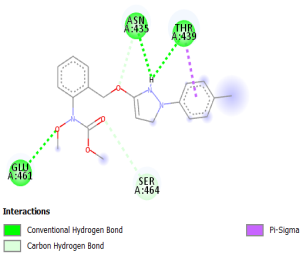 | 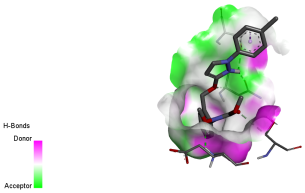 |
| 5_L019_pythopthora  _1_PyRx_AutoDock4 error | 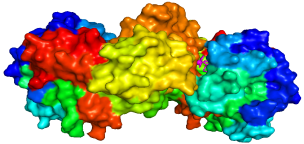 | 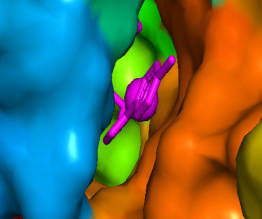 | 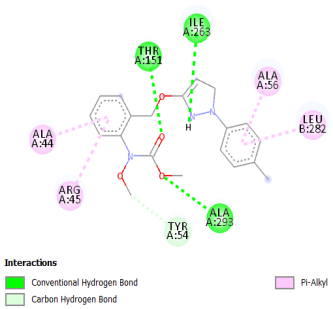 | 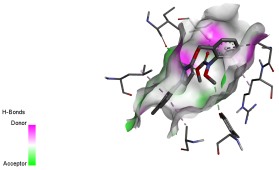 |
| 5_L019_Sclerotium  _2_PyRx_AutoDock4 error | 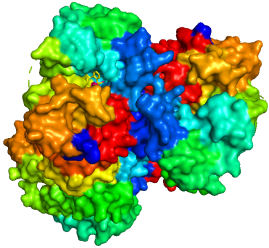 | 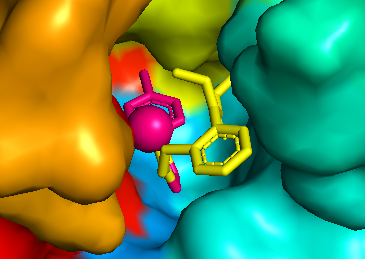 | 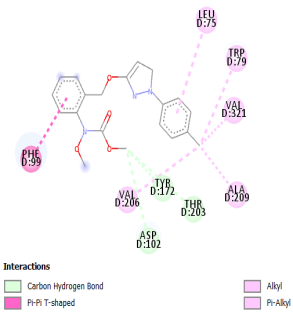 | 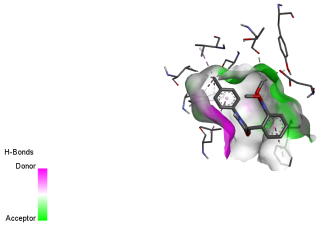 |
