# Supplementary material for: Computational and In silico study of novel fungicides against combating root rot, gray mold, fusarium wilt, and cereal rust
Source: PLoS One. 2025 Jan 31;20(1):e0316606. doi: 10.1371/journal.pone.0316606 (PMC11785347; doi:10.1371/journal.pone.0316606)
Supplement: Attachment — Submitted filename: Reviewer Comments_PONE-D-24-06911.docx [file pone.0316606.s006.docx]

The paper presents a comprehensive computational study on the identification of potential fungicides against four fungal proteins. The authors employ a range of computational methods, including molecular docking, molecular dynamics simulations, and pharmacokinetics studies. However here are some suggestions

1. Title should be shortened and more concise
2. Should add relevant keywords that accurately reflect the content of the paper.
3. Don’t use jargon in title
4. Add description or full form of these proteins i.e. 7VEM, 8H6Q, 8EBB, 7XDS, atleast at first time use
5. Also add sequence characterization study for the given proteins
6. Add details of figure in legends
