# Supplementary material for: Computational and In silico study of novel fungicides against combating root rot, gray mold, fusarium wilt, and cereal rust
Source: PLoS One. 2025 Jan 31;20(1):e0316606. doi: 10.1371/journal.pone.0316606 (PMC11785347; doi:10.1371/journal.pone.0316606)
Supplement: Attachment — Submitted filename: Reviewer Comments_PONE-D-24-06911 (R2).docx [file pone.0316606.s007.docx]

The paper presents a comprehensive computational study on the identification of potential fungicides against four fungal proteins. The authors employ a range of computational methods, including molecular docking, molecular dynamics simulations, and pharmacokinetics studies. However here are some suggestions

1. **Title should be shortened and more concise.**

*Answer: It has revised and have made short as*

*“Computational and In silico study of Novel Fungicides against Combating Root Rot, Gray Mold, Fusarium Wilt, and Cereal Rust.”*

2. **Should add relevant keywords that accurately reflect the content of the paper.**

*Answer: It has revised and added as*

Density Functional Theory, HOMO, LUMO, Molecular docking, ADMET and Molecular Dynamics

3. **Don’t use jargon in title**

*Answer: It has revised and sincerely removed.*

4. **Add description or full form of these proteins i.e. 7VEM, 8H6Q, 8EBB, 7XDS, at**

**least at first time use.**

*Answer: It has revised and added as:*

The proteins 7VEM, 8H6Q, 8EBB, and 7XDS, derived from various devastating plant pathogens, represent critical targets for computational drug discovery aimed at combating agricultural diseases. The protein 7VEM, identified as NADPH-assisted quinone oxidoreductase from *Phytophthora capsici*, plays a crucial role in maintaining redox balance essential for the pathogen's survival and virulence, making it a prime target for inhibitor design. Solved at 2.39 Å resolution with an R-work value of 0.167, its structural fidelity and lack of mutations ensure reliable insights for virtual screening and molecular docking. Similarly, the Class I sesquiterpene synthase BCBOT2 (apo) from *Botrytis cinerea* (PDB ID: 8H6Q), resolved at 2.00 Å with R-free and R-work values of 0.195 and 0.168 respectively, is vital for sesquiterpene biosynthesis linked to the pathogen's virulence. Its structural data offer a robust framework for identifying small molecules to disrupt sesquiterpene synthesis, attenuating *B. cinerea*'s pathogenicity. The SIX6 protein from *Fusarium oxysporum* f. sp. lycopersici (PDB ID: 8EBB), despite its classification as a protein of unknown function, plays a critical role in the virulence mechanism of this vascular wilt pathogen, which devastates crops like tomatoes. Its high-resolution structure at 1.88 Å, with R-free and R-work values of 0.221 and 0.193, supports molecular docking, virtual screening, and lead optimization to design antifungal agents. Finally, the AvrSr35 effector protein from *Puccinia graminis* *f.* sp. tritici (PDB ID: 7XDS), resolved at 2.06 Å with reliable R-values (R-free: 0.287, R-work: 0.257, Observed: 0.258), provides critical insights into wheat stem rust pathogenicity. Its potential as a target for structure-based drug design is underscored by its well-defined active sites and suitability for molecular dynamics simulations and virtual screening. Together, these structural models offer a comprehensive platform for advancing the design of inhibitors to mitigate plant pathogen-induced diseases Table 1.

**Table 1. Protein information.**

| Title | PDB ID:7VEM | PDB ID:8H6Q | PDB ID:8EBB | PDB ID:7XDS |
| --- | --- | --- | --- | --- |
| Organism | *Phytophthora capsici* | *Botrytis cinerea* | *Fusarium oxysporum* f. sp.*lycopersici* | *Puccinia graminis* f. sp. *tritici* |
| Resolution | 2.39 Å | 2.00 Å | 1.88 Å | 2.06 Å |
| R-Value Free | 0.224 | 0.195 | 0.221 | 0.287 |
| Ramachoron plot, % | 89.5 | 93.8 | 91.8 | 94.7 |

5. **Also add sequence characterization study for the given proteins**

*Answer: It has revised and added which is almost relevant answer of question 4.*

6. **Add details of figure in legends**

Answer: *Answer: It has revised and added as:*

The study of a computational procedure to determine the quantum calculations of any chemical species requires the optimization of the molecular structure, which is an important aspect of its structural geometry.

Additionally, accurate computational parameters are obtained by determining the most stable configuration of any chemical structure. In this study, all compounds underwent computational optimization using the DFT functional, and their primary and most stable configuration was observed with minimal energy required for optimization. The antifungal ligands are Azoxystrobin(L01), Cyproconazole(L02), Difenoconazole (L03), Tebuconazole (L04), Tricyclazole (L05), Chlorothalonil (L06), Benalaxyl (L07),Bismerthiazol (L08), Carbendazim (L09), Hexaconazole (L10), Thiram (L11), Carboxin (L12), Iprodione (L13), Kresoxim-Methyl (L14), Cymoxanil (L15), Dichloran (L16), Propiconazole (L17), Dimethomorph (L18), Pyraclostrobin (L19) andAmetoctradin (L20) are shown in **Fig 1 and supplementary 1.**

|  |  |
| --- | --- |
| L01 | L02 |
|  |  |
| L03 | L04 |
