# Supplementary material for: Computational and In silico study of novel fungicides against combating root rot, gray mold, fusarium wilt, and cereal rust
Source: PLoS One. 2025 Jan 31;20(1):e0316606. doi: 10.1371/journal.pone.0316606 (PMC11785347; doi:10.1371/journal.pone.0316606)
Supplement: Attachment — Submitted filename: comments.docx [file pone.0316606.s005.docx]

Introduction: Poorly written introduction with false information and old references. Also, most of the description is without any reference. For example, author has not added any reference for first paragraph and justified the statement with myco website link. Reference have been added here for mycodata bank. Second reference is from the study conducted in 2006 which does not justify author’s statement “Approximately 20,000 various species of plants have been impacted by fungus related………….”. Third or seventh reference is again from 90s era which is not updated one to quote the statement.

Methods and Results: Only structure-based study without any sequence based data cannot interpret the interaction between fungicide and four fungal pathogens. For docking, single software/tool provides limited knowledge. Author should have used multiple software to evaluate its results. Result section has several inaccurate terms to describe the details. For example, binding energy or Gibbs free energy was described as a docking result throughout the article. In Methods, 8H6Q is mentioned as *B. cinerea* PDB and then the result section says it’s as *S. sclerotiorum*. Figure legends are without proper caption or details. Figure 5 is not mentioned in the description. Figures are added in low resolution. Beta factor is described without any reference in result section and figure 13 is without any details in the text. No idea what figure 13 shows.

There is not discussion section to highlight worth of this study in correspondence to past 5 years data on these pathogens. No references were added for in-silico studies.
